# Fresh Rumen Liquid Inoculant Enhances the Rumen Microbial Community Establishment in Pre-weaned Dairy Calves

**DOI:** 10.3389/fmicb.2021.758395

**Published:** 2022-01-12

**Authors:** Hanna Huuki, Seppo Ahvenjärvi, Paula Lidauer, Milka Popova, Johanna Vilkki, Aila Vanhatalo, Ilma Tapio

**Affiliations:** ^1^Department of Agricultural Sciences, University of Helsinki, Helsinki, Finland; ^2^Production Systems, Genomics and Breeding, Natural Resources Institute Finland (Luke), Jokioinen, Finland; ^3^Production Systems, Animal Nutrition, Natural Resources Institute Finland (Luke), Jokioinen, Finland; ^4^Production Systems, Welfare of Farmed Animals, Natural Resources Institute Finland (Luke), Jokioinen, Finland; ^5^Institute National de la Recherche Agronomique, UMR 1213 Herbivores, Clermont Université, VetAgro Sup, UMR Herbivores, Clermont-Ferrand, France

**Keywords:** microbiome manipulation, microbiome establishment, dairy calf, ciliate protozoa, anaerobic fungi, bacteria, archaea, rumen function

## Abstract

The development of the functional rumen in calves involves a complex interplay between the host and host-related microbiome. Attempts to modulate rumen microbial community establishment may therefore have an impact on weaning success, calf health, and animal performance later in life. In this experiment, we aimed to elucidate how rumen liquid inoculum from an adult cow, provided to calves during the pre-weaning period, influences the establishment of rumen bacterial, archaeal, fungal, and ciliate protozoan communities in monozygotic twin calves (*n* = 6 pairs). The calves were divided into treatment (T-group) and control (C-group) groups, where the T-group received fresh rumen liquid as an oral inoculum during a 2–8-week period. The C-group was not inoculated. The rumen microbial community composition was determined using bacterial and archaeal 16S ribosomal RNA (rRNA) gene, protozoal 18S rRNA gene, and fungal ITS1 region amplicon sequencing. Animal weight gain and feed intake were monitored throughout the experiment. The T-group tended to have a higher concentrate intake (Treatment: *p* < 0.08) and had a significantly higher weekly weight gain (Treatment: *p* < 0.05), but no significant difference in volatile fatty acid concentrations between the groups was observed. In the T-group, the inoculum stimulated the earlier establishment of mature rumen-related bacterial taxa, affecting significant differences between the groups until 6 weeks of age. The inoculum also increased the archaeal operational taxonomic unit (OTU) diversity (Treatment: *p* < 0.05) but did not affect the archaeal quantity. Archaeal communities differed significantly between groups until week 4 (*p* = 0.02). Due to the inoculum, ciliate protozoa were detected in the T-group in week 2, while the C-group remained defaunated until 6 weeks of age. In week 8, *Eremoplastron dilobum* was the dominant ciliate protozoa in the C-group and *Isotricha* sp. in the T-group, respectively. The Shannon diversity of rumen anaerobic fungi reduced with age (Week: *p* < 0.01), and community establishment was influenced by a change of diet and potential interaction with other rumen microorganisms. Our results indicate that an adult cow rumen liquid inoculum enhanced the maturation of bacterial and archaeal communities in pre-weaning calves’ rumen, whereas its effect on eukaryotic communities was less clear and requires further investigation.

## Introduction

The microbial ecosystem inhabiting the rumen enables ruminants to utilize plant materials as feed. However, immediately after birth, ruminant feed digestion more resembles that of monogastrics (Comline and Titchen, 1951; Baldwin et al., 2004; Meale et al., 2017). It takes several weeks of morphological, physiological, and metabolical changes, induced by the ontogenic regulation, nutrition, and establishment of rumen microorganisms, for the rumen to become fully functional to provide for most of the animal’s energy needs (Baldwin et al., 2004; Khan et al., 2016; Meale et al., 2017; Malmuthuge et al., 2019). The successful development of functional rumen during the pre-weaning period may affect animal performance later in life (Yáñez-Ruiz et al., 2015). Understanding how this system can be influenced is therefore one of the current research focal points worldwide.

Active gut colonization of newborns starts at birth through exposure to the microbiomes of the dam’s vagina and skin, feces, mouth, colostrum, and milk (Yeoman et al., 2018; Klein-Jöbstl et al., 2019), as well as through the housing environment, with other conspecifics (Bird et al., 2010). During the pre-ruminant phase (0–14 days), when calves are sustained on a liquid diet, the rumen function is still very low, as most of the liquid is digested by the enzymes in the abomasum and small intestine (Comline and Titchen, 1951; Ørskov et al., 1970; Baldwin et al., 2004). Nevertheless, during this period, the rumen starts being populated by the aerobic and facultative anaerobic bacteria, gradually changing to obligate anaerobes (Jami et al., 2013; Rey et al., 2014). Cellulolytic and proteolytic bacteria, necessary for a functional rumen, have been detected in the rumen as early as 20 min to 3 days after birth (Li et al., 2012; Rey et al., 2012, 2014; Jami et al., 2013; Guzman et al., 2015). Methanogenic archaea also start populating the rumen during the first days of life (Guzman et al., 2015; Friedman et al., 2017), while anaerobic fungi have been observed in the rumen at around 1 week after birth (Fonty et al., 1987).

In the transition phase (from 2 weeks to weaning), solid feed particles and liquid start entering the rumen, fueling the fermentation process and causing physical stress that promotes the expansion of the volume and muscular development of the rumen and rumen motility (Khan et al., 2016). During this period, the papillae’s growth increases the rumen epithelium surface area needed for more efficient volatile fatty acid (VFA) absorption and metabolism (Baldwin et al., 2004; Khan et al., 2016; Meale et al., 2017). Rumination starts at around 2 weeks of age, and after 6 weeks, it gradually reaches a level similar to that of adult cows (Swanson and Harris, 1958). The transition phase is related to further rearrangements of microbial community composition (Dias et al., 2017), with ciliate protozoa being the last microorganisms to colonize the rumen. They have been detected in animals after 2 weeks of age, once stable bacterial community and rumen conditions have been established (Eadie, 1962b; Fonty et al., 1988).

Attempts to affect early life rumen microbial colonization and development have explored various strategies. Natural rearing with the mother affects the microbiota development (Abecia et al., 2017; Belanche et al., 2019b) and can have a positive impact on young ruminants’ performance (Belanche et al., 2019a). Dietary intervention studies have demonstrated that supplementing the liquid diet with concentrate (Malmuthuge et al., 2013; Jiao et al., 2015; Dias et al., 2017) or forage (Jiao et al., 2015; Carballo et al., 2019; Dill-McFarland et al., 2019) can promote rumen maturation and alter the rumen microbial community. The use of antimicrobials has been found to alter microbial communities and reduce methane emissions (Abecia et al., 2013; Meale et al., 2021). Similarly, the inoculation of calves with rumen liquid influences their rumen bacterial community composition by developing into more adult-like, improved papillae growth and increases dry matter intake (Cox et al., 2019). In lambs and goats, a rumen inoculum has improved growth performance (Zhong et al., 2014; Belanche et al., 2020), dry matter intake (De Barbieri et al., 2015; Belanche et al., 2020), and microbial colonization during the pre-weaning period (Belanche et al., 2020; Palma-Hidalgo et al., 2021), while no effect on calf growth and microbial composition was observed when bacteria- or protozoa-enriched inoculum was administered (Cersosimo et al., 2019).

Despite research efforts, the mechanisms of how rumen microbial community development can be modulated remain elusive. The genetics of an individual plays a role, as monozygotic or dizygotic twins have more similar microbiota than non-related individuals in humans (Turnbaugh et al., 2009; Goodrich et al., 2014, 2016; Koo et al., 2019) and calves (Mayer et al., 2012). In this study, we therefore used monozygotic twin calves to reduce the potential variation in microbial community establishment caused by the differences in animals’ genetic background. Our goal was to modulate rumen microbial community colonization in calves during the pre-weaning period by inoculating them with fresh rumen liquid obtained from a cow with a low residual energy intake value (Mäntysaari et al., 2012). We assessed the effect of inoculation on the establishment of rumen general and core microbiome and rumen fermentation characteristics. We hypothesized that the microbial community structure of treated calves would more closely resemble that of the adult cow and that the inoculum might promote fermentation processes in the rumen and potentially stimulate the growth of calves.

## Materials and Methods

### Animals and Experimental Design

Fifteen Nordic Red dairy cows were impregnated with twin embryos produced by embryo splitting (Herr and Reed, 1991) at the Natural Resources Institute Finland (Luke). Six pregnancies resulted in the calving of twins (July 23–October 28, 2017). After birth, the calves were left with their dam for an hour and then separated into individual pens (147 cm × 172 cm) that prevented physical contact and microbial exchange between the calves or contact with other animals throughout the experiment. Pens were furnished with straw bedding, a heat lamp, a basket for hay, and buckets for water and concentrate. The calves were weighed at birth and within each pair were randomly assigned to either an inoculation treatment (T-group) or a control group (C-group).

The calves from both groups were managed and fed in the same way. Within 2 h of birth, all the calves received 2 L of the same high-quality (Brix value 24) colostrum mix as the first meal to ensure uniformity in the passive transfer of immunity *via* the colostrum. Colostrum from 3 cows was collected in advance, aliquoted into 0.5-L portions, and stored at −20°C until use. Before use, portions were warmed to 40°C, pooled, and bottle-fed to the calves. As the second meal, the twins received their own dam’s colostrum from the first milking and thereafter were fed bulk colostrum. From day (d)4 onward, they received 2 L of milk four times a day. From d8, the milk was gradually replaced with the milk replacer (Startti Maito Instant, Valio, Finland). Weaning was carried out gradually, starting on d29. The calves were completely weaned from the milk replacer after the experiment by d57. The detailed schedule of the weaning process is presented in Supplementary Table 1. Hay and calf concentrate (Pikku-mullin-herkku, Raisio Agro, Finland) were offered from d1 and topped up according to consumption. Starting from week 7, grass silage was offered up to a maximum of 2 kg/day. The dietary ingredients and chemical composition of feeds are presented in Supplementary Tables 2, 3.

Starting from week 2, the calves in the T-group received rumen liquid obtained from a fistulated multiparous cow (176–269 days in milk) fed a concentrate:forage diet (ratio 48:52) and previously identified as a feed-efficient animal based on a low residual energy intake (Mäntysaari et al., 2012). Freshly collected rumen liquid was given to the calves orally through a silicone tube attached to a syringe. During weeks 2 and 3, the oral dose was 5 ml/day, given 3 times a week (Monday, Wednesday, and Friday). Later, in week 4, the dose was increased to 10 ml/day. The inoculation treatment ended when the calves were 8 weeks old.

The calves’ health was monitored and recorded daily by the farm veterinarian. During the treatment period, two calves from both groups were offered electrolyte (Benfital Plus nutrient supplement, Boehringer Ingelheim Danmark A/S) for diarrhea, and two calves in the T-group and one in the C-group received a dose of activated carbon paste (Lehmän HIILI-pasta, FinnCow, Finland). Animal weight was measured at birth and once a week thereafter. The weekly weight gain was calculated by subtracting the previous week’s weight from the current weight.

Feed refusals were recorded after each meal to measure the intake of liquid diet, concentrate, and grass silage. Daily feed intake was calculated by subtracting the weight of leftovers from that of the offered feed. Hay was only monitored by recording the offered amount of hay per day. Real consumption was impossible to measure due to the hay falling onto the straw bedding. The solid feed intake was reported as g of dry matter based on the feed analysis.

### Sample Collection

Blood samples were collected from the jugular vein in K2E EDTA tubes (Greiner Bio-one, Austria) once a week during the 8-week period. Analysis of blood immunoglobulin G (IgG) was performed at Movet oy (Kuopio, Finland) to confirm the proper transfer of passive immunity.

Rumen fluid was collected *via* the esophageal 1-m polyvinyl chloride (PVC) tube (9/13 mm inner/outer diameter) into a glass jar attached to a manual vacuum pump (Ruminator^1^, Germany). The samples were collected 3 h after morning feeding in weeks 2, 4, 6, and 8 at least 24 h after the previous inoculation. Immediately after collection, 500-μl aliquots were snap-frozen in dry ice and stored at −80°C until DNA extraction.

The rumen sample collection for VFA and ammonia-N determination was carried out as described by Ahvenjärvi and Huhtanen (2018). Briefly, fresh rumen liquid was filtered through two layers of cheesecloth, a 5-ml aliquot was mixed with 0.5 ml of saturated mercuric (II) chloride solution and 2 ml of 1 M sodium hydroxide solution, and stored at −20°C for later VFA analysis with gas chromatography (Huhtanen et al., 1998). To determine the ammonia-N concentrations, 15 ml of rumen liquid was mixed with 0.3 ml of 50% sulfuric acid and stored at −20°C for later analysis based on the direct colorimetric method (McCullough, 1967).

To assess the rumen inoculum microbial community composition, 500-μl samples from each inoculum batch were collected, snap-frozen in dry ice, and stored at −80°C until DNA extraction. Before DNA extraction, samples from each week were pooled into 16 samples, so that they represented the different weeks throughout the treatment period.

### DNA Extraction, Library Preparation, and Sequencing

The total DNA was extracted from 500 μl of rumen liquid samples, as described by Rius et al. (2012), and stored at −20°C for further analysis. Libraries of the bacterial and archaeal 16S ribosomal RNA (rRNA) V4 region were prepared following the “16S metagenomics sequencing library preparation” protocol (Illumina) using 515F and 806R primers (Caporaso et al., 2011) with Illumina adapters. The same protocol was adapted for the library preparation of the ciliate protozoa 18S rRNA V3 region using 316F and 539R primers (Sylvester et al., 2004) and for the internal transcribed spacer 1 (ITS1) region of rumen anaerobic fungi using Neo18SF and Neo5.8SR primers (Edwards et al., 2008). A detailed description of primers is provided in Supplementary Table 4, and protocols for library preparation are in Supplementary Material 1. Libraries were sequenced on Illumina MiSeq (Finnish Functional Genomics Centre, Turku) using the Paired-End approach and 2 × 250 bp chemistry for the 16S rRNA gene library and 2 × 300 bp for the ciliate protozoa 18S rRNA and fungal ITS1 libraries. The number of samples for each animal group and week that successfully amplified are presented in Table 1.

**TABLE 1:** Alpha-diversity estimates for bacterial, archaeal, ciliate protozoan, and fungal communities in treatment (T-group) and control (C-group) calves during the 8-week experimental period.

Study week	Group	Bacteria				Archaea				Ciliate protozoa				Anaerobic fungi			
		*N*	Observed OTUs	Shannon diversity	Simpson	*N*	Observed OTUs	Shannon diversity	Simpson	*N*	Observed OTUs	Shannon diversity	Simpson	*N*	Observed OTUs	Shannon diversity	Simpson
**Week 2**	**T-group**	6	482 ± 133	5.20 ± 0.50	0.92 ± 0.03	6	8 ± 4	1.70 ± 0.60	0.59 ± 0.12	5	73 ± 9	2.20 ± 0.30	0.69 ± 0.06	5	438 ± 107	4.00 ± 0.50	0.88 ± 0.03
	**C-group**	6	411 ± 41	5.30 ± 0.40	0.94 ± 0.02	5	2 ± 1	0.50 ± 0.40	0.18 ± 0.17	0	–	–	–	6	467 ± 87	4.40 ± 0.40	0.91 ± 0.02
**Week 4**	**T-group**	6	963 ± 315	7.00 ± 0.90	0.97 ± 0.02	6	15 ± 4	2.30 ± 0.50	0.69 ± 0.10	6	84 ± 11	2.50 ± 0.60	0.74 ± 0.10	3	440 ± 41	4.30 ± 0.00	0.90 ± 0.01
	**C-group**	6	597 ± 88	6.11 ± 0.30	0.97 ± 0.01	6	8 ± 2	1.50 ± 0.40	0.48 ± 0.12	0	–	–	–	6	392 ± 111	3.60 ± 1.00	0.81 ± 0.14
**Week 6**	**T-group**	6	1,063 ± 516	7.20 ± 1.30	0.97 ± 0.02	6	16 ± 8	2.10 ± 0.70	0.61 ± 0.17	6	89 ± 11	2.40 ± 0.90	0.67 ± 0.25	3	431 ± 117	4.10 ± 0.50	0.88 ± 0.04
	**C-group**	6	732 ± 93	6.60 ± 0.50	0.97 ± 0.01	6	9 ± 3	1.60 ± 0.60	0.52 ± 0.21	3	82 ± 24	2.20 ± 1.10	0.60 ± 0.33	4	313 ± 115	3.00 ± 1.10	0.71 ± 0.24
**Week 8**	**T-group**	6	1,015 ± 601	6.80 ± 1.60	0.96 ± 0.03	6	13 ± 6	2.00 ± 0.40	0.62 ± 0.07	6	74 ± 18	1.90 ± 1.20	0.50 ± 0.30	2	328 ± 33	2.80 ± 0.10	0.64 ± 0.08
	**C-group**	6	837 ± 200	6.60 ± 0.70	0.96 ± 0.03	6	13 ± 6	1.70 ± 0.20	0.53 ± 0.13	4	76 ± 19	1.60 ± 1.20	0.42 ± 0.32	4	252 ± 158	2.40 ± 0.80	0.61 ± 0.14
***p*-value**	**Wk**		<0.001	<0.001	0.002		0.005	0.072	0.207		0.131	0.369	0.414		0.048	0.007	0.003
	**Trt**		0.228	0.421	0.757		0.015	0.003	0.001		0.642	0.499	0.554		0.543	0.238	0.320
	**Trt × Wk**		<0.001	0.002	0.019		0.002	0.009	0.012		0.437	0.737	0.796		0.212	0.012	0.011

*The number of samples for each group and week is indicated as N. Data are presented as averages ± standard deviation. The statistical significance of Treatment (Trt), Week (Wk), and Treatment by Week interaction (Trt × Wk) was tested with the Kruskal–Wallis rank sum test. The C-group remained defaunated until week 6. The statistical analyses for ciliate protozoa between the groups were performed for week 6 and week 8 data only. OTU, operational taxonomic unit.*

### Sequence Data Processing

Sequence data demultiplexing, adaptor removal, and sorting of sequences by barcode were performed by the sequencing provider (Finnish Functional Genomics Centre, Turku). Sequencing data were further processed using Qiime v 1.9.1 (Caporaso et al., 2010). Briefly, the forward and reverse reads were joined using SeqPrep and filtered by quality (>Q20) and length (279–300 bp). Filtered sequences were clustered into operational taxonomic units (OTUs) at 97% similarity using UCLUST (Edgar, 2010). Chimeric reads from bacteria data were removed using ChimeraSlayer (Haas et al., 2011) and from fungal and ciliate protozoa data using usearch61 (Edgar, 2010). The taxonomy of the bacterial OTUs was assigned using Greengenes (gg 13 8 otus) (DeSantis et al., 2006), the archaeal using RIM-DB (Seedorf et al., 2014), ciliate protozoan using ciliate protozoa (Kittelmann et al., 2015), and fungal OTUs using ITS (Koetschan et al., 2014), reference databases. Singleton OTUs were removed. The prokaryote data were evenly subsampled to 17,000, ciliate protozoa to 18,000, and fungal data to 25,000 reads/sample before further analysis to reduce the depth heterogeneity. The sufficient sequencing depth was confirmed by the rarefaction plots (Supplementary Material 1 and Supplementary Figure 1). The sequence reads are available in the Sequence Read Archive (SRA) BioProject PRJNA713003.

### Quantification of Microbial Communities

The quantities of bacteria, archaea, ciliate protozoa, and anaerobic fungi were estimated with qPCR by quantifying rRNA gene copy numbers of each taxonomic group in 1 ng of extracted DNA. The bacteria were quantified by amplifying the 16S rRNA gene V4 area (279 bp) using primers 520F and 799r2cor (Supplementary Table 4). Amplification reaction (20 μl) contained 0.75 × TB Green Premix Ex Taq II (TaKaRa Bio Inc., China), 0.5 μM of each primer, and 20 ng of DNA. The amplification was done in a StepOnePlus thermocycler (Applied Biosystems, Villebon-sur-Yvette, France) with denaturation at 95°C for 30 s, 40 cycles of denaturation at 95°C for 15 s, and annealing at 60°C for 30 s. For archaea quantification, the 16S rRNA gene (510 bp) was amplified using 896F and 1406R primers (Supplementary Table 4). The reaction was carried out at a volume of 10 μl, with a 1 × Power SYBR green PCR master mix (Applied Biosystems by Thermo Fisher Scientific, Life Technologies Ltd., United Kingdom), 0.15 μM of each primer, and 10 ng of template DNA. The quantification of archaea was done in a Viia7 thermocycler (Applied Biosystems) with initial denaturation at 95°C for 10 min, 40 cycles of denaturation at 95°C for 15 s, and annealing at 60°C for 1 min. Ciliate protozoa were quantified by amplifying the 18S rRNA gene region (223 bp) using 316F and 539R primers (Supplementary Table 4). Amplification was carried out at a volume of 10 μl, with a 1 × Power SYBR green PCR master mix (Applied Biosystems by Thermo Fisher Scientific, Life Technologies Ltd., United Kingdom), 0.25 μM of each primer, and 10 ng of template DNA. The quantification was done in a Viia 7 thermocycler (Applied Biosystems) with initial denaturation at 95°C for 10 min, 40 cycles of denaturation at 95°C for 30 s, annealing at 60°C for 30 s, and extension at 72°C for 30 s. Anaerobic fungi were quantified by amplifying the ITS1 region (120 bp) with FungiF and FungiR primers (Supplementary Table 4). The reaction was carried out at a volume of 15 μl, with a 1 × Power SYBR green PCR master mix (Applied Biosystems by Thermo Fisher Scientific, Life Technologies Ltd., United Kingdom), 2 mM of MgCl_2_, 1 μM of each primer, and 10 ng of template DNA. The quantification of anaerobic fungi was done in a Viia7 thermocycler (Applied Biosystems) with initial denaturation at 95°C for 10 min, 45 cycles of denaturation at 95°C for 15 s, and annealing at 60°C for 1 min. For bacteria and archaea, the melt curve was performed with denaturation at 95°C for 15 s, annealing at 65°C for 15 s, and final denaturation at 95°C for 15 s, with a ramp increment of 0.4°C. For ciliate protozoa and fungi, the melt curve annealing temperature was set to 60°C for 1 min, followed by final denaturation at 95°C for 15 s, with a ramp increment of 0.4°C. All standards and samples were analyzed with three replicates. The absolute quantities of experimental samples were estimated against serial dilutions of DNA standards ranging from 10^2^ to 10^9^ copies per reaction. A description of the standard preparation is provided in Supplementary Material 1. The results were regressed against the logarithmic scale concentration to achieve the standard curve and sample quantities.

### Statistical Analyses

The rumen VFA and ammonia-N concentrations, bacterial and archaeal 16S rRNA gene copies, average weekly feed intake, weight gain, and blood IgG levels were analyzed using the mixed procedure, with a repeated approach in SAS version 9.4 (SAS Institute Inc., Cary, NC, United States). Normality was confirmed with the Shapiro–Wilk test. Weight, concentrate, and silage intake data were log-transformed to achieve normal distribution and later reverse transformed for data interpretation. The calf was treated as the experimental unit. Treatment, Week, and Treatment × Week interaction were treated as fixed effects. Pair and Pair × Week interaction were treated as random effects. The fact that samplings were repeated over time and were therefore correlated was taken into account by modeling the correlations between weeks and week variances using appropriate covariance structures selected based on model fit statistics. The effects were estimated using the Residual Maximum Likelihood (REML) method and were declared significant at *p* ≤ 0.05. Pairwise comparisons between Week, Treatment, and Treatment × Week interaction were performed using Tukey’s test. The copy numbers of ciliate protozoa 18S rRNA gene and fungal ITS1 regions were analyzed with the non-parametric Kruskal–Wallis rank sum test and pairwise comparisons with the Wilcoxon rank sum test.

Microbial community alpha diversity changes in the T- and C-groups over the 8-week period were estimated using the Shannon index, the Simpson index, and the number of observed OTUs, as implemented in Qiime. The statistical analysis was performed using the Kruskal–Wallis rank sum test and pairwise comparisons with the Wilcoxon rank sum test.

To explore the treatment and time effect on the changes in the microbial community structure, between-sample diversity was evaluated as Bray–Curtis dissimilarities following the Hellinger transformation and visualized using principal coordinate analysis (PCoA), as implemented in R packages *Microbiome* (Lahti et al., 2017) and *Phyloseq* (McMurdie and Holmes, 2013). The significance for the Week, Treatment, and Treatment × Week interaction was estimated using distance-based permutational multivariate analysis (Adonis) as implemented in the R package *vegan* (Oksanen et al., 2019) and the pairwise comparisons between the Treatment × Week interaction with permutational multivariate analysis of variance with the false discovery rate adjustment with the R package *RVAideMemoire* (Herve, 2020). Treatment and week effects on individual microbial taxa in the T- and C-groups were evaluated using the ANOVA and mixed procedure as described above. OTUs with relative abundance below 0.1% and detected in less than 6 samples were filtered out from the bacterial and fungal datasets. To achieve normal distribution and deal with 0-values, data were [log_2_(1 + *x*)] transformed prior to further analysis. Treatment, Week, and the Treatment × Week interaction were treated as fixed effects, and pairwise comparisons were made using Tukey’s test.

To explore core microbiome establishment in the T- and C-groups during different stages of development, the core microbiome was defined as a set of OTUs that was present in all calves within a group at a particular week tested. The impact of inoculation on the core microbiome establishment in calves was evaluated by the proportion of OTUs shared between the donor and calves. A more detailed description of statistics is given in Supplementary Material 1.

## Results

### Passive Immunity and General Health

**FIGURE 1:**
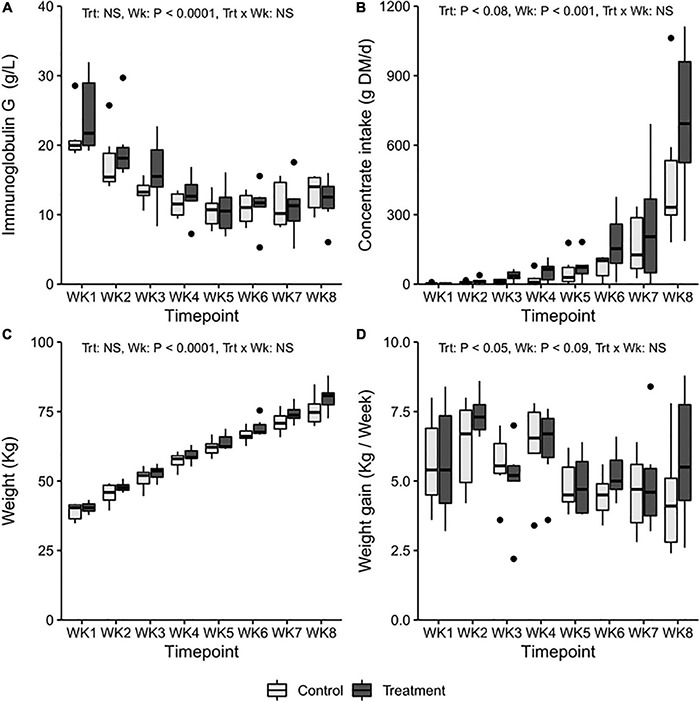
The average weekly **(A)** blood immunoglobulin G levels, **(B)** average daily concentrate DM intake, **(C)** weekly weight (kg), and **(D)** average weight gain in treatment (T-group, dark gray) and control group (C-group, light gray) calves during the 8-week treatment period.

At the beginning of the experiment, blood IgG levels indicated a sufficient passive transfer of IgG after birth, and the concentration was similar in both groups (Figure 1A). No significant changes related to the initiation of inoculation were observed in the IgG levels in the T-group at the age of 2 weeks (Treatment: *p* > 0.05, Treatment × Week: *p* > 0.05). The mean IgG levels dropped from a “high” (>24 g/l) in the T-group and “medium” (16–24 g/l) in the C-group to a “low” level (16–8 g/l) in week 3 (Week: *p* = 0.0001) and further declined until week 5, after which they stabilized.

The calves in both groups remained in good general health throughout the experiment. Four calves in the T-group and six calves in the C-group had an incidence of diarrhea that occurred between 2 and 4 weeks of age. One calf from the C-group and two calves from the T-group also had an elevated body temperature associated with diarrhea.

### Feed Intake and Growth

The consumption of colostrum, milk, and milk replacer was similar in both groups throughout the experiment (Supplementary Table 5). The preferences for different solid feeds varied individually. The T-group tended to eat more concentrates [32.6 ± 1.6, least square means (LSM) ± standard error (SE) g dry matter (DM)/day] than the C-group (17.6 ± 1.6 g DM/day) across the entire treatment period (Treatment: *p* = 0.08) (Figure 1B), but the difference was not significant when weekly consumption by group was compared (Treatment × Week: *p* > 0.05).

There were no significant differences in mean weight between the groups at birth (T-group: 34.8 ± 1.5; C-group: 33.4 ± 1.5, LSM ± SE kg). During the following weeks, the mean body weight in the T-group was numerically higher than that in the C-group (Treatment: *p* > 0.05, Week: *p* < 0.01, Treatment × Week: *p* > 0.05) (Figure 1C). The T-group gained an average of 350 g more weight per week across the treatment period (Treatment: *p* < 0.05) (Figure 1D).

### Volatile Fatty Acids

**FIGURE 2:**
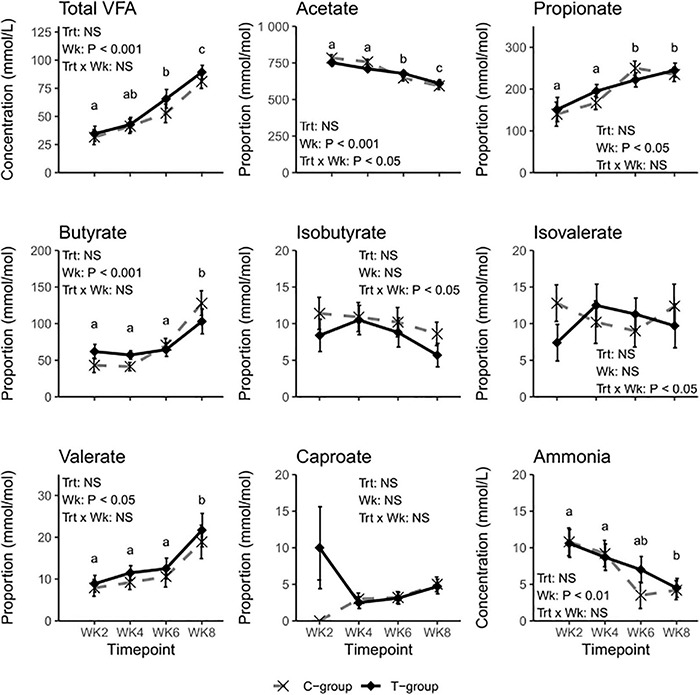
Rumen fermentation characteristics of treatment (T-group, black solid line) and control group (C-group, gray dashed line) calves during the 8-week treatment period. Data for individual volatile fatty acids (VFAs) are presented as molar proportions of total VFA. The significance of Treatment (Trt), Week (Wk), and interaction (Trt × Wk) effects is presented in the figure. The different letters indicate significant differences in the pairwise comparisons of sampling weeks.

The total ruminal VFA concentration increased with age in both groups but was unaffected by the treatment (Figure 2). Week had an effect on most of the individual VFAs. In both groups, the molar proportion of acetate decreased (Week: *p* < 0.01), while propionate, butyrate, and valerate increased (Week: *p* < 0.05) during the 2–8-week period. A significant Treatment × Week interaction indicated a greater decline of acetate molar proportions in the C-group. The molar proportions of isobutyrate, isovalerate, or caproate were unaffected by the week (Week: *p* > 0.05). Although significant Treatment × Week interactions were observed for isobutyrate and isovalerate, there were no significant differences between the groups. The proportion of ammonia reduced toward the end of the pre-weaning period (Week: *p* < 0.01) but was not significantly affected by the treatment.

### Microbial Community Quantities

**FIGURE 3:**
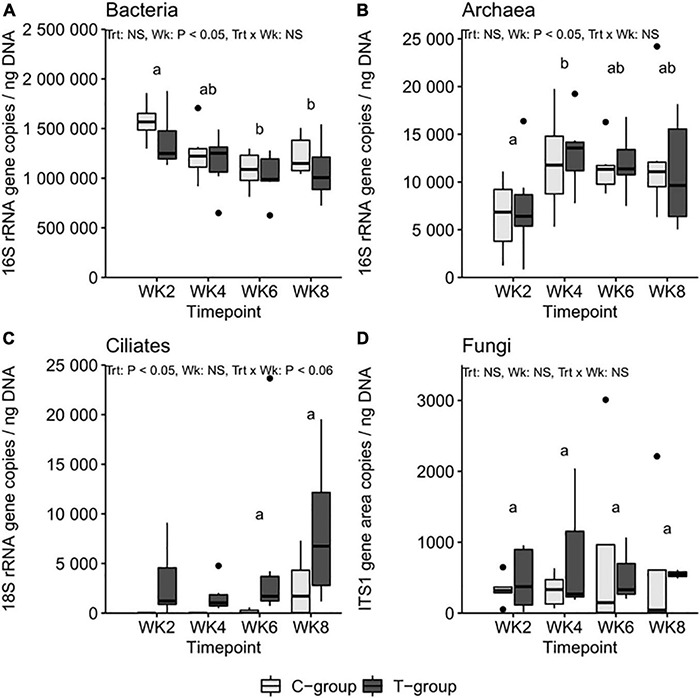
The **(A)** bacteria 16S ribosomal RNA (rRNA), **(B)** archaea 16S rRNA, **(C)** ciliate protozoa 18S, and **(D)** anaerobic fungi internal transcribed spacer 1 (ITS1) region copy numbers per ng of rumen DNA in treatment (T-group) and control (C-group) calves during the 8-week treatment period. The significance of Treatment (Trt), Week (Wk), and interaction (Trt × Wk) effects is presented in the figure. Letters indicate significant differences in the pairwise comparisons of sampling weeks. The C-group remained defaunated until week 6: the statistical tests for rumen ciliate protozoa were therefore performed only for week 6 and week 8 data.

The treatment had no significant effect on the 16S rRNA gene copy numbers for total bacteria or archaea (Treatment: *p* > 0.05). Nevertheless, the number of bacterial 16S rRNA gene copies was highest in both groups at 2 weeks of age and decreased toward the end of the pre-weaning period, being at its lowest in week 6 (Figure 3A). The number of copies in archaea was lowest in week 2 (Week: *p* < 0.05), doubled in concentration in week 4, and remained stable thereafter (Figure 3B). The treatment significantly increased the ciliate protozoa 18S rRNA gene copy numbers (Treatment: *p* < 0.05), but due to high variance, no significant differences between the groups were observed (Figure 3C). The number of samples no longer observed to have anaerobic fungi increased toward week 8 (Table 1), but the copy numbers of the ITS1 region within samples that amplified remained at a similar level throughout the experiment and were unaffected by the treatment (Figure 3D).

### Sequencing

After quality filtering, the data from 48 calf samples contained 1,329,850 (mean 27,705 ± 5,183) prokaryote reads, and the data from 16 donor pooled inoculum samples contained 807,724 (50,483 ± 9,060) prokaryote reads. From them, 19,891 (414 ± 239) reads in the calves and 42,585 (2,662 ± 631) in the donor cow were identified as archaeal. The ciliate protozoan data from the calves contained 2,499,249 (83,308 ± 189,856) reads and from the donor contained 1,267,635 (79,227 ± 6,967) reads. The fungal data from the calves contained 2,266,229 (68,674 ± 16,942) reads and the donor contained 1,359,617 (84,976 ± 13,091) reads in total.

#### Bacteria

The treatment had no significant effect on bacterial alpha diversity. The observed number of OTUs, Shannon diversity, and Simpson’s evenness were lowest in both groups in week 2 and increased over the pre-weaning period (in all *p* < 0.05; Table 1).

**FIGURE 4:**
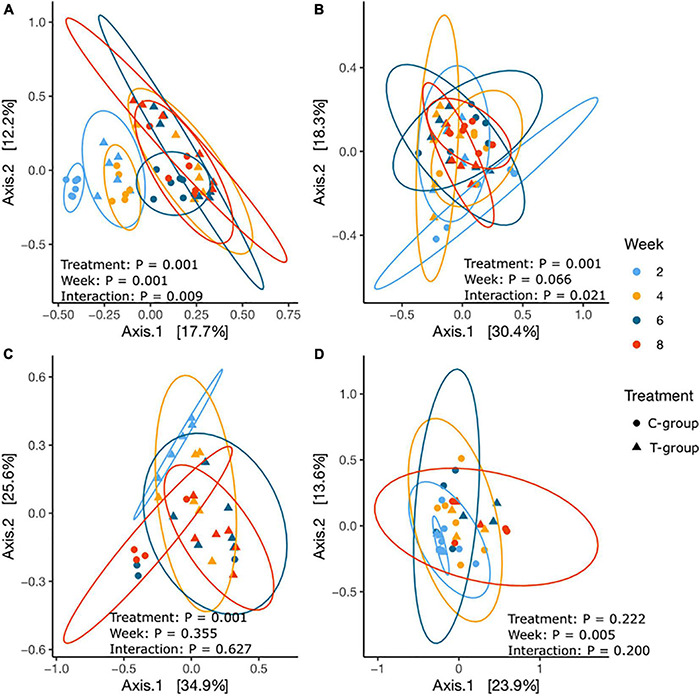
The principal coordinate analysis of Bray–Curtis dissimilarities for **(A)** bacterial, **(B)** archaeal, **(C)** ciliate protozoan, and **(D)** anaerobic fungal communities in treatment (T-group, ▲) and control (C-group, •) calves at 2 weeks (light blue), 4 weeks (yellow), 6 weeks (dark blue), and 8 weeks (red) of age.

Beta diversity calculated as Bray–Curtis dissimilarities significantly differentiated samples by Week (*p* = 0.001), Treatment (*p* = 0.001), and Treatment × Week interaction (*p* = 0.009) (Figure 4A and Supplementary Table 6). Week 2 samples in both groups were significantly separated from the later weeks, and the bacterial community structure remained significantly different between the T- and C-groups until week 6. Within the T-group, no significant differences were observed after week 4; in the C-group, after week 6.

**FIGURE 5:**
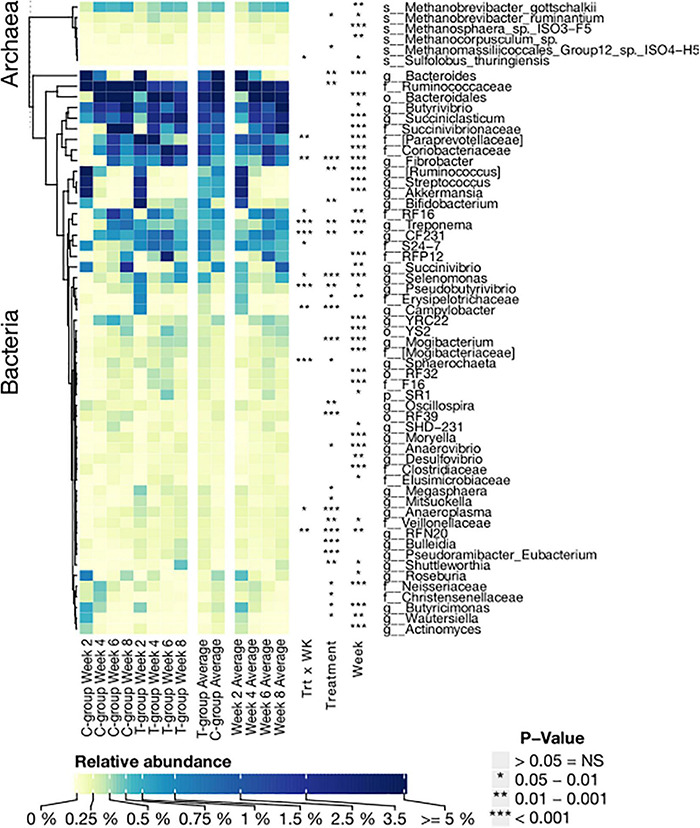
The relative abundance of significantly affected bacterial genera and archaeal species in treatment (T-group) and control group (C-group) calves during the 8-week treatment period. Data are presented as averages of treatment by week, averages of treatment across the dataset, and averages of week across the dataset. The annotation on the right indicates the significance level of Treatment (Trt) and Week (Wk) and their interaction (Trt × Wk). The letter in front of the taxon name indicates the closest identified taxonomic level: species (s); genus (g); family (f); order (o); and phylum (p).

The number of bacterial phyla increased from 12 to 17 over the pre-weaning period, but only Firmicutes, Spirochaetes, and Fibrobacteres were significantly affected by the treatment (Supplementary Table 7). To better understand bacterial colonization, we grouped bacterial taxa based on their timewise abundance trend in the C-group. We examined the taxa associated with liquid feed digestion, which naturally diminished toward weaning, and taxa associated with solid feed fermentation, which increased in abundance with age and saw differences in colonization dynamics between the groups (Figure 5 and Supplementary Table 8). As a result of inoculation, in week 2, *Fibrobacter*, *Treponema*, CF231, *Selenomonas*, *Sphaerochaeta*, *Pseudobutyrivibrio*, *Anaerovibrio*, *Veillonellaceae* spp., and RFN20 were only observed or were significantly more abundant in the T-group calves. These taxa were also among the most abundant (>0.1%) taxa in the donor samples (Supplementary Table 8). At the same time, *Bacteroides*, *Wautersiella*, *Butyricimonas*, genus [*Ruminococcus*] in the family *Lachnospiraceae*, and *Neisseriaceae* spp., which were low abundant in the donor, decreased in abundance earlier in the T-group than the C-group calves (Figure 5 and Supplementary Table 8). Several taxa such as *Campylobacter* and a [*Paraprevotellaceae*] genus in the order Bacteroidales increased in abundance toward the end of the pre-weaning period in the C-group, whereas in the T-group, their abundances diminished after week 2. A set of bacterial taxa showed no clear age-dependent variation, but its abundance was affected by the treatment. *Bifidobacterium*, *Megasphaera*, and *Mogibacterium* were significantly more abundant in the T-group, whereas the C-group was significantly enriched in *Oscillospira*, *Ruminococcaceae* spp., *Christensenellaceae* spp., and RF39. Where the other aforementioned taxa were also abundant in the donor, *Megasphaera* and *Campylobacter* were low. Nevertheless, in week 8, most of the significant differences between the groups disappeared (Figure 5 and Supplementary Table 8).

#### Archaea

The number of observed archaeal OTUs was lowest in both groups in week 2 and increased significantly over time (*p* < 0.05), but the T-group had numerically or significantly (week 4; *p* < 0.05) more OTUs. The Shannon diversity was significantly higher in the T-group in week 2 (*p* < 0.01), with no differences observed between the groups in later weeks (Table 1).

The Bray–Curtis dissimilarities indicated that the archaeal community structure differed between the T- and C-groups until week 4 (*p* < 0.05), but no longer thereafter. Within the T-group, the archaeal community showed no significant differences between weeks (all *p* > 0.05), whereas in the C-group, the community in week 2 differed significantly from week 8 (*p* < 0.05), suggesting a gradual development (Figure 4B and Supplementary Table 6).

Twenty archaeal species were identified in the entire dataset (Supplementary Table 9). In week 2, calves in the T-group had a taxonomically more diverse archaeal community dominated by *Methanobrevibacter gottschalkii*, *Methanobrevibacter ruminantium*, and *Methanosphaera ISO3-F5*, which were also dominant in the donor samples (Figure 5 and Supplementary Table 9). *Methanomicrobium mobile*, *Methanocorpusculum parvum*, and several *Methanomassiliicoccales* species were also present in lower abundances in the T-group. In contrast, in the C-group in week 2, not all the calves were colonized with archaea. The dominant *Mbb. gottschalkii* was detected in five calves; two had *Mbb. ruminantium* and *Methanosphaera ISO3-F5*, while *M. mobile* was detected in one animal. *Methanomassiliicoccales* and *Methanocorpusculum* colonized the rumen of the C-group calves after week 4 or later. The abundance of *Mbb. gottschalkii* and *Methanosphaera* sp. *ISO3-F5* increased toward the end of the pre-weaning period (Week: *p* < 0.01), while *Mbb. ruminantium* reached its highest values in week 4 in both groups (Week: *p* < 0.05). Inoculation in the T-group increased the relative abundance of *Mmc.* Group12 sp. ISO4-H5 and *Mbb. ruminantium* (Treatment: *p* < 0.05) (Figure 5 and Supplementary Table 9).

#### Ciliate Protozoa

The ciliate protozoa alpha diversity, calculated for weeks 6 and 8, was unaffected by the treatment, and the number of observed OTUs, the Shannon index, and Simpson evenness remained at a similar level in both groups (Week: *p* > 0.05, Table 1).

**FIGURE 6:**
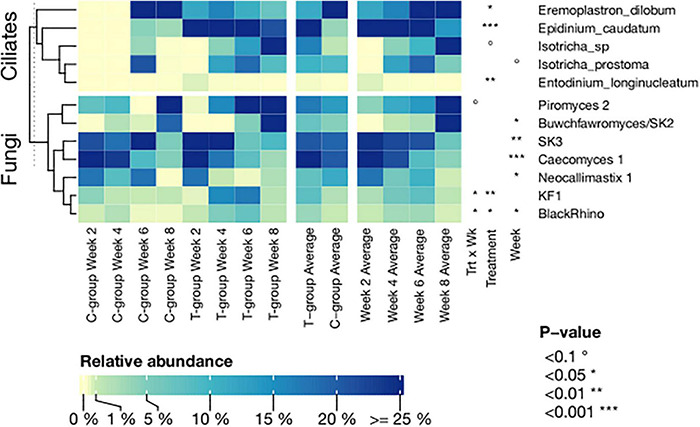
The relative abundance of significantly affected ciliate protozoan and fungal species in treatment (T-group) and control group (C-group) calves during the 8-week treatment period. Data are presented as averages of treatment by week and averages of treatment and week across the dataset. The annotation on the right indicates the significance level of the Treatment (Trt) and Week (Wk) and their interaction (Trt × Wk).

The beta diversity analysis significantly differentiated the T-group ciliate protozoan community in week 2 from the succeeding weeks (*p* = 0.001), but after week 4, the communities no longer differed (Figure 4C and Supplementary Table 6). In the C-group, ciliate protozoa were detected in three animals in week 6 and in four animals in week 8, and the community compositions did not differ significantly (*p* > 0.05). However, significant differences were observed between the C- and T-groups in week 8 (Treatment: *p* = 0.001) (Figure 6 and Supplementary Table 10).

In total, 25 genera- and 40 species-level ciliate protozoan taxonomic groups were identified in the entire dataset (Supplementary Table 10). Donor samples were largely dominated by *Epidinium caudatum*, and apart from few very rare observations, all species observed in both calf groups were also observed in donor samples. In week 2, the T-group was dominated by *E. caudatum*, followed by *Eremoplastron dilobum*, and *Entodinium* sp., which together accounted for 90% of the abundance data (Figure 6 and Supplementary Table 10). In week 4, *Isotricha prostoma* also colonized the rumen and continued to increase in abundance during the following 4 weeks, becoming the dominant species in the T-group in week 8. In contrast, the ciliate protozoan community in the C-group in weeks 6 and 8 was dominated by *E. dilobum*. *I. prostoma* was the second most abundant species in week 6, but in week 8, it was replaced by *Entodinium furca monolobum* (Figure 6 and Supplementary Table 10).

#### Anaerobic Fungi

The fungal alpha diversity decreased over time, and no significant differences between the groups were observed (Treatment: *p* > 0.05) (Table 1). During the later sampling weeks, several samples from both groups failed to produce fungal amplicons.

Beta diversity analysis showed that the fungal communities in week 2 differed from those of later weeks (*p* < 0.05), but there were no significant differences between the T- and C-groups in any sampling week (all: *p* > 0.05) (Figure 4D and Supplementary Table 6).

In total, 18 species-level fungal groups were observed in the dataset (Supplementary Table 11). All taxa observed in both calf groups were also observed in the donor community, which was dominated by *Neocallimastix 1*, SK3, and *Piromyces* 2. In week 2, both the C- and T-groups were dominated by *Caecomyces 1*, SK3, and *Neocallimastix 1.* Over the following weeks, *Caecomyces 1*, *Neocallimastix 1*, and SK3 declined in abundance, while *Piromyces* 2 and *Buwchfawromyces/SK2* became dominant in both groups in week 8. Inoculation significantly increased the abundance of *BlackRhino* (Treatment: *p* < 0.05) in week 4 and KF1 (Treatment: *p* < 0.01) in week 6 in the T-group (Figure 6 and Supplementary Table 11). In week 8, the T- and C-groups differed numerically in the abundance of *Piromyces* 1, which was 16.2% in the C-group and 0.03% in the T-group, but was nearly absent (0.002%) in the donor. However, the fungal abundances were affected by large inter-animal variation (Supplementary Table 11).

### Core Microbiome

To better understand if there was a limited set of taxa that was important for rumen microbial maturation, and if this set could be influenced by the inoculum, we investigated the core microbiome differences between the groups. Due to continuous changes in rumen microbial colonization over the pre-weaning period, a core microbiome was defined for each week and group separately. Only OTUs present in 100% of animals in a particular group in a particular week were included in the core.

#### Core Bacterial and Archaeal Community Development

Each sampling week had a distinct bacterial core, but we evaluated the speed of maturation by the extent of core OTUs that remained in the core over the subsequent weeks. An earlier establishment of mature microbiome-related taxa was already observed in week 2 in the T-group calves, as 27% of core OTUs in the T-group, compared to 5% in the C-group, were shared with the core in week 8 (Supplementary Table 12).

**FIGURE 7:**
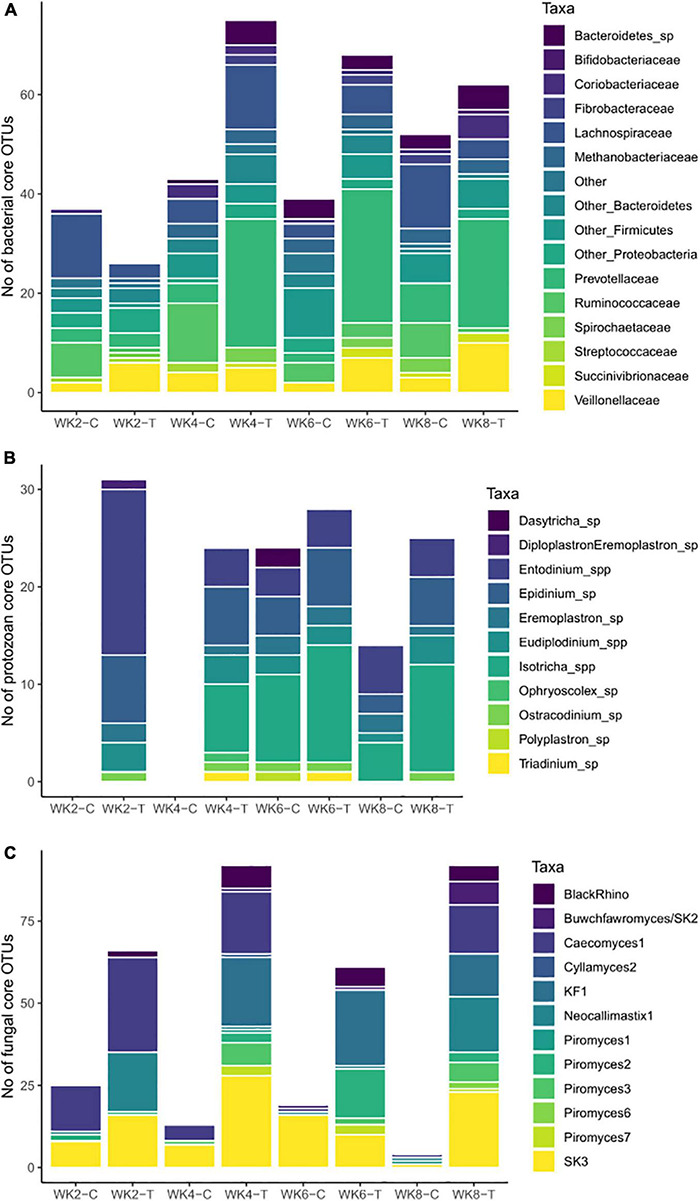
The number of operational taxonomic units (OTUs) and taxonomic composition of **(A)** the core prokaryote community (archaea and bacteria), **(B)** the core ciliate protozoan community, and **(C)** the core anaerobic fungal community in treatment (T) group and control (C) group calves at 2 (WK2), 4 (WK4), 6 (WK6), and 8 (WK8) weeks of age.

The core bacterial community was composed of 26–78 OTUs, which increased with age and represented an average of 30% of the total bacterial abundance. To a substantial extent, the core OTUs belonged to Bacteroidetes, Firmicutes, Proteobacteria, Actinobacteria, and Euryarchaeota phyla, but the number of OTUs per phylum and the OTUs’ affiliation at family and genus levels differed between the groups (Figure 7A and Supplementary Table 12). As a result of inoculation, the number of core OTUs in the T-group tripled between weeks 2 and 4 and remained at an elevated level throughout the rest of the experiment. The increase in core OTUs was caused by the considerable number of *Prevotella* OTUs entering the core, and the dominance of *Prevotella* within Bacteroidetes remained over the following weeks. On the other hand, throughout the whole pre-weaning period, the C-group retained a larger share of Firmicutes core OTUs than the T-group, but the taxonomic composition at family level was age- and group-specific (Supplementary Table 12). In the C-group, *Ruminococcaceae* and *Lachnospiraceae* families were dominant in the core in all weeks, while the *Lachnospiraceae* and *Veillonellaceae* families dominated the core in the T-group. The core archaeal OTUs belonged to the *Methanobrevibacter*, *Methanosphaera*, and *Methanomassiliicoccaceae* genera, and their presence in the core was group- and week-specific. The T-group core had *Methanobrevibacter* from week 2, while the C-group had *Methanobrevibacter* only from week 4. The donor cow inoculum had an impact on the establishment of the bacterial core because 16 core OTUs in week 2, 76 OTUs in week 4, and all core OTUs in week 8 were common between the T-group calves and the donor (Supplementary Table 12). Proteobacteria-affiliated core OTUs alone were mostly not obtained from the donor, but the genera detected were age-specific. *Succinivibrionaceae* OTUs were observed in the core of the T-group from week 4, while in the C-group, they were observed only in week 8 (Figure 7A).

#### Core Ciliate Protozoan Community Development

The ciliate protozoan core community was more uniform across sampling weeks and represented an average of 94% of the total protozoan abundance. The number of shared OTUs with week 8 in the T-group increased from 42% in week 2 to 75% in week 6. In the C-group, 46% of OTUs were shared between weeks 6 and 8. However, in both groups, the total number of core OTUs decreased with age (Figure 7B). Core OTUs affiliated with the *Entodinium*, *Epidinium*, *Diploplastron/Eremoplastron*, *Eudiplodinium/Eremoplastron*, and *Ostracodinium* genera populated the T-group in week 2, while *Isotricha* joined the core community after week 4 (Supplementary Table 13). In the C-group, the taxonomic composition of the core in week 6 was similar to the T-group, with the exception of *Polyplastron multivesiculatum* and *Dasytricha ruminantium*. In week 8, the core ciliate protozoa were shared between both calf groups and the donor (Supplementary Table 13).

#### Core Fungal Community Development

There were substantial differences in the anaerobic fungal core community development between the groups (Figure 7C). The core in the T-group comprised an average of 83% of the total fungal abundance, while the core in the C-group represented an average of only 38%, suggesting larger inter-animal variation in the C-group calves and a more stable core community composition in the T-group. The effect of inoculation was already observed in week 2, when the T-group calves had 3 times more core OTUs than similar-aged C-group animals, and 59% of these OTUs were shared with the core in week 8 (Supplementary Table 14). The effect of inoculation was also observed in the taxonomic diversity. In week 2, both groups’ fungal core was dominated by *Caecomyces 1*, *SK3*, and *Neocallimastix 1.* While the fungal taxonomical core in the C-group remained similar over the pre-weaning period, the T-group core in week 4 had OTUs affiliated with 11 genera, with *KF1*, *Buwchfawromyces/SK2*, *Cyllamyces 2*, and *Piromyces 1*, *3*, and *7* joining the core (Figure 7C and Supplementary Table 14). The taxonomic composition of the T-group core after week 4 remained similar. Nearly all OTUs observed in the T- and C-groups were present in the donor, although some were of low counts.

## Discussion

In this study, we used monozygotic twin calves and tested the effect of fresh mature rumen liquid inoculum on rumen microbial community development during the pre-weaning period. Several studies have demonstrated positive effects on phenotypic characteristics and gut development in pre-weaned ruminants (Muscato et al., 2002; Zhong et al., 2014; Cox et al., 2019; Belanche et al., 2020; Palma-Hidalgo et al., 2021) caused by rumen liquid inoculation, but in bovines, the results are inconclusive (Cersosimo et al., 2019; Bu et al., 2020; Yu et al., 2020). The differences in research outcomes may be related to high between-host variation (Mayer et al., 2012). While the host effect can be controlled in twin or triplet experiments in small ruminants (e.g., Belanche et al., 2020; Palma-Hidalgo et al., 2021), twin calving in dairy livestock is rare. In the present study, monozygotic twin calves were therefore created to reduce the genetic host variation. Our results indicate that inoculation enhanced the bacterial and archaeal community establishment and induced differences in ciliate protozoan community composition, but despite continuous inoculation, it was unable to promote the establishment of a rumen anaerobic fungi community.

### Animal Growth and Rumen Fermentation

The inoculum promoted better weight gain in the T-group animals, which was probably associated with an increase in concentrate intake after 6 weeks of age. Our results are in line with those of other studies, which have reported a positive effect of fresh or autoclaved rumen liquid on animals’ weight gain (Zhong et al., 2014; Belanche et al., 2020). It is known that butyrate, but also propionate and acetate, even in small doses, stimulates rumen papillae development and rumen epithelia metabolism and absorption (Sander et al., 1959; Sakata and Tamate, 1978; Suárez et al., 2006; Guilloteau et al., 2009; Connor et al., 2014). We therefore hypothesized that the inoculum could stimulate rumen fermentation processes in T-group calves and increase VFA production. However, the better weight gain in the T-group animals in this study could not be explained by the rumen fermentation differences because the VFA profiles between the groups were very similar. The lack of significant differences in VFA proportions was probably a reflection of the lack of a significant difference in the total microbial quantities between the groups. As many microbes can produce the same VFA end products, the VFA proportions were unaffected by the differences between the groups in the rumen microbial community composition.

In contrast with the observations by Rey et al. (2012), in which VFA concentration stabilized around 1 month of age, we observed an increase in VFA concentration in both groups until the end of the experiment. The discrepancies between the studies may be explained by the differences in dietary composition. In the present study, silage was added to the diet in week 7 and may have stimulated fermentation processes in the calves’ rumen. The steeper trend in the reduction of acetate in the C-group between weeks 4 and 6 may be a result of increasing concentrate intake, but it also coincides with the first observations of the ciliate protozoa in the C-group calves. The absence of ciliate protozoa has previously been associated with a slight increase in acetate concentration (Newbold et al., 2015). However, due to the small sample size and high interindividual variation, pairwise comparisons failed to show any significant differences between the groups.

### Effect of Inoculum on Bacteria Community Development

Immediately after birth, the rumen bacterial community gradually changes from facultative anaerobic bacteria to obligate anaerobes (Jami et al., 2013; Rey et al., 2014). Similarly, in the present study, at 2 weeks of age, the bacterial communities in both calf groups differed from the succeeding weeks. Week 2 bacteria were represented by a broad range of sugar utilizers such as *Bacteroides* (Wexler, 2014), aerobes, or facultative anaerobes, e.g., *Neisseriaceae* spp. (Adeolu and Gupta, 2013) and *Streptococcus* (Stewart et al., 1997), which later reduced in abundance. Moreover, there were also bacteria with special niches among them, such as *Akkermansia* and *Ruminococcus*, capable of utilizing oligosaccharides from milk, mucosa, and saliva (Derrien, 2007; Huang et al., 2011; Bell et al., 2019; Kostopoulos et al., 2020) or being associated with the epimural community (Stewart et al., 1997; Derrien, 2007; Jami et al., 2013; Malmuthuge et al., 2014; Jiao et al., 2015). As the microenvironments during the early life within the rumen change rapidly, many of these taxa reduced in abundance or disappeared earlier from the T-group animals, suggesting that the environment within the T-group rumen was changing more quickly. Our observations are in line with a previous finding in which a faster reduction of several early life-related taxa and the promotion of rumen function and development could be induced with dietary measures (Dias et al., 2017).

In the T-group animals, inoculation stimulated the earlier establishment of taxa related to mature rumen function and fiber degradation, e.g., *Treponema* or the fibrolytic genus *Fibrobacter* (Shinkai et al., 2010; Jewell et al., 2015). The abundance of these taxa increased especially noticeably during the first 4 weeks of life. The positive impact of inoculation was also already visible in the composition of the T-group core community in week 4. The presence of a large number of OTUs in the core, which pertained throughout the rest of the experiment, suggests that the conditions within the T-group calf rumen were sufficient for these mature rumen-related bacterial OTUs to become established and remain functional. Our results support the recent findings of Palma-Hidalgo et al. (2021), who reported that a similar rumen liquid inoculum induced positive effects on rumen bacteria establishment in goats.

We also hypothesized that rumen microbial maturation was related to the reduced Bacteroidetes taxonomic diversity and increased taxonomic diversity of Firmicutes within the rumen. Indeed, in the core communities of both groups, the number of OTUs assigned to the *Prevotella* (Avguštin et al., 1997) genus increased substantially with age, and it became the dominant taxon, replacing nearly all other OTUs assigned to Bacteroidetes. This increase in *Prevotella* occurred earlier (week 4) in the T-group than the C-group (week 8). It has previously been suggested that the relative abundance of *Prevotella* increases with age as a result of increasing fiber content in the diet (Jami et al., 2013; Rey et al., 2014). However, other observations are inconclusive (Li et al., 2012; Dias et al., 2018; Cersosimo et al., 2019). Members of the genus *Prevotella* are broad-range carbohydrate utilizers, and a considerable fluctuation in metabolic capabilities can be expected between species or even between strains within the same species. It is therefore best to evaluate the role of *Prevotella* at the OTU level. It is possible that, as versatile fermenters, *Prevotella* spp. work as hubs in the network of microbial interactions and help maintain the community’s interactions. On the other hand, the inoculum treatment resulted in differences in the dominant taxa within the Firmicutes core community. Although the major fiber-degrading taxa were present in the core of both groups in week 8, the T-group had more OTUs assigned to *Veillonellaceae*, whereas the C-group had more OTUs assigned to the *Ruminococcaceae* and *Lachnospiraceae* families. These differences may be related to the competitive advantages provided by the continuous inoculation and availability of feed substrates.

### Effect of Inoculum on Archaea Community Development

We hypothesized that by receiving archaea with the inoculum from an adult cow, and with ciliate protozoa and fungi present in the community, hydrogen sources for methanogenesis would be present in T-group animals, creating suitable conditions for hydrogenotrophic archaea growth. However, the treatment did not significantly increase the 16S rRNA gene copy numbers of total archaea. It is possible that factors other than the availability of hydrogen limited the archaea’s growth in the developing rumen because the methanogenic community of young calves is known to utilize, e.g., methylamines and methanol for methanogenesis more efficiently than an adult methanogenic community (Friedman et al., 2017). Nevertheless, the inoculum treatment preponed the archaea establishment and increased archaea richness and differences in community composition during the first 4 weeks. Our results are in line with the previous observations by Dias et al. (2017) showing that *Mbb. gottschalkii* is present in milkfed calves, while the abundance of *Methanosphaera* increases with the provision of concentrate. *Methanobrevibacter* is the dominant clade of methanogens in the rumen and uses the hydrogenotrophic pathway to produce methane, whereas methylotrophic *Methanosphaera* can use other substrates such as methanol and methylamines (Morgavi et al., 2010), and may require a more diverse microbial community to produce them. Similarly, in line with the previous results of Friedman et al. (2017), the establishment of *Methanomassiliicoccales* appeared later, suggesting that this archaea might benefit from the more mature microbial community, providing a substrate for energy metabolism. The differences in the archaea richness and community composition between the T- and C-groups during the experiment’s first weeks suggest that the archaea from the donor could remain in the rumen possibly due to the more mature microbial community in the T-group, which was able to provide the conditions for a more diverse archaeal community. However, despite the later establishment of archaeal communities in the C-group, by the end of the liquid feeding period, the archaeal composition in both groups was very similar.

### Effect of Inoculum on Ciliate Protozoan Community Development

Protozoa are naturally established in the rumen at around 15–30 days of age (Eadie, 1962b; Naga et al., 1969; Cersosimo et al., 2019) and require contact between conspecifics for transmission (Bird et al., 2010). Given that all the calves in the present experiment were kept in individual pens to prevent physical contact, our results suggest that the first adult rumen inoculum was already successful in providing viable ciliate protozoa to the T-group’s calves. However, half of the C-group calves also had ciliate protozoa by week 6. Despite no close proximity, the pens were located in a barn where other cows were also housed, so microbial transmission through aerosol droplets in the farm air or manual transmission through unrecorded mistakes in management practices cannot be excluded. Moreover, the calves remained with the dam for a short period after birth, and the dam may have transferred some ciliate protozoa through saliva (Tapio et al., 2016), which was established later, when conditions in the rumen became favorable. With real-time quantitative PCR methods, protozoa have been detected as early as in 1-day-old goats (Abecia et al., 2014), making this hypothesis plausible.

The treatment significantly increased the ciliate protozoan quantity in the T-group animals, estimated with 18S rRNA gene copy numbers. The establishment of a ciliate protozoan community requires a well-functioning bacterial population to be in place (Fonty et al., 1988), and it can further be affected by the inoculum’s cell density (Williams and Dinusson, 1972), the type and amount of feed (Kudo et al., 1990; Franzolin and Dehority, 1996; Santra and Pathak, 2001), the pH, and other conditions of the rumen (Eadie, 1962b; Dehority, 2005; Franzolin and Dehority, 2010). The increase in the quantity of rumen ciliates in the T-group may therefore have been affected by several of these factors. Although the ciliate protozoa in the T-group underwent taxonomic changes within the 2–4-week period, these changes did not follow the sequential establishment previously observed in young ruminants, in which *Entodinium* spp. were among the first species to establish, followed by *Diplodinium* and Holotrichs (Bryant et al., 1958; Eadie, 1962b; Naga et al., 1969; Minato et al., 1992; Dias et al., 2017). In the T-group in week 2, *Epidinium* was the dominant protozoan, accounting for 70% of total abundances. Interestingly, it was also the dominant genus in the donor. However, the *Epidinium* decreased in T-group over time due to an increase in the abundance of *Isotricha*, while the *Entodinium* level remained constant. It can be hypothesized that the inoculum provided a significant number of protozoan colonizers, which had well-established community interactions and required less time for reestablishment. The increase in concentrate intake and presence of silage in the diet at the end of the pre-weaning period may also have promoted the rumen microbiome’s overall function, thus improving the ciliate community’s growth.

Due to different exposures to external microbiome sources, the C-group calves in week 8 were dominated by the *Diploplastron/Eremoplastron* genus. Previous studies have shown that the A-type and B-type members in a stable ciliate community rarely coexist due to predation and competition for resources (Eadie, 1962a,^1967^). While the T-group community included B-type genera like *Eudiplodinium*, *Epidinium*, and *Eremoplastron* obtained from the donor cow, the A-type *P. multivesiculatum* was detected in the C-group core community, suggesting a different seeding source. We hypothesize that the C-group acquired the ciliate protozoan community in a more random manner, requiring several selection steps, which finally led to a community dominated by different taxa than those of the donor cow and the T-group calves.

### Effect of Inoculum on Anaerobic Fungi Community Development

The frequent provision of the inoculum did not directly improve the establishment of rumen anaerobic fungi. Although nearly all the calves had fungi at the beginning of the experiment, only two T-group calves and four C-group calves were observed to have fungi at 8 weeks. The decreasing observations of fungi after the beginning of a solid feed diet have previously been described (Fonty et al., 1987; Dias et al., 2017; Dill-McFarland et al., 2019). The authors speculated that during weaning, as the solid feed intake increased and the competition of carbohydrate sources intensified, the fungal community had to reestablish itself by occupying a different niche in plant structural carbohydrate breakdown. Due to a slower life cycle, the fungi may not have been able to compete for substrate and may have started to diminish. In our study, both groups received the same diet; therefore, other factors may have played a role in the fungal community being more present in the C-group than the T-group. For example, inhibition or predation by other microbial groups may have promoted the vanishment of fungi. Bacteria have been observed to inhibit fungal growth and cellulase activity (Dehority and Tirabasso, 2000), and protozoa are known to be able to ingest and digest fungal zoospores (Morgavi et al., 1994; Miltko et al., 2014). Lower predation pressure would explain why the fungi only started to disappear in the C-group at 6 weeks of age, when the ciliate communities started emerging. Furthermore, fermentation products and changes in pH can influence fungal community development (Joblin and Naylor, 1993; Srinivasan et al., 2001).

Inoculation influenced the taxonomic composition of rumen anaerobic fungi. In line with Dias et al. (2017), *Caecomyces 1* and SK3 dominated the fungal communities of milkfed calves, but fungal taxa underwent changes during the 4–8-week period, when the solid feed intake started to increase. Although the major fungal taxa were similar between the groups, *BlackRhino* and KF1 were more specific to the T-group, while *Piromyces 1* was more abundant in the C-group, possibly indicating different seeding sources, as *BlackRhino* and KF1 were observed in the donor, but *Piromyces 1* only in negligible abundance. Gordon and Phillips (1998) suggested that fungi are probably transferred to the calf through direct contact with the mother or contaminated feeds. This is supported by evidence that fungi have been isolated from the saliva and feces of ruminants (Tapio et al., 2016), and they can be cultivated after prolonged exposure to oxygen, drought, or freezing temperatures (Milne et al., 1989; Davies et al., 1993; McGranaghan et al., 1999) probably due to the production of resistant spores (Brookman et al., 2000).

The positive influence of the inoculum in the T-group animals was observed in the richness of the fungal core community. While the core of the C-group in week 8 was reduced to only 4 OTUs, the core community of the T-group contained 92 OTUs, indicating wide fungal taxonomic diversity in the rumen. It is tempting to speculate that a less diverse fungal community could survive better through the pre-weaning period. As shown previously, a less diverse microbiome may be more efficient because it reduces the various metabolic pathways and interactions between taxa (Kruger Ben Shabat et al., 2016). However, high interindividual variation and the decreasing number of study subjects observed to have fungi may have affected our observations. More research is needed to assess the effects of weaning on the development of the anaerobic fungal community in pre-weaning calves.

## Conclusion

Our results show that the orally administered microbial inoculum successfully reached the rumen and induced changes in the microbial community structure during the pre-weaning period. However, the inoculum affected the different microbial groups differently: the bacteria and archaea communities matured more quickly than the control calves, and the inoculum improved the establishment of ciliate protozoa, whereas fungal colonization was poor and probably occurred later. Inoculation with adult rumen liquid stimulated weight gain and tended to improve solid feed intake in the T-group during the pre-weaning period. However, these differences between the groups could not be explained by the rumen fermentation parameters.

## Data Availability Statement

The datasets presented in this study can be found in online repositories. The names of the repository/repositories and accession number(s) can be found below: https://www.ncbi.nlm.nih.gov/bioproject/PRJNA713003.

## Ethics Statement

The animal study was reviewed and approved by the National Ethics Committee (ESAVI/5687/04.10.07/2017, Hämeenlinna, Finland).

## Author Contributions

IT and JV designed the study. IT, PL, and SA conducted the animal experiment and sample collection. HH, MP, and IT performed the laboratory work. HH, SA, and IT analyzed the data. HH and IT wrote the manuscript. All authors read and approved the final manuscript.

## Conflict of Interest

The authors declare that the research was conducted in the absence of any commercial or financial relationships that could be construed as a potential conflict of interest.

## Publisher’s Note

All claims expressed in this article are solely those of the authors and do not necessarily represent those of their affiliated organizations, or those of the publisher, the editors and the reviewers. Any product that may be evaluated in this article, or claim that may be made by its manufacturer, is not guaranteed or endorsed by the publisher.

## Footnotes

1
profs-products.com
