# Supplementary material for: Fresh Rumen Liquid Inoculant Enhances the Rumen Microbial Community Establishment in Pre-weaned Dairy Calves
Source: Front Microbiol. 2022 Jan 12;12:758395. doi: 10.3389/fmicb.2021.758395 (PMC8790516; doi:10.3389/fmicb.2021.758395)
Supplement: Supplementary file 1 [file Data_Sheet_1.zip › Supplementary Material 1.docx]

Supplementary Material 1

# Materials and methods

## Library preparation and sequencing

Libraries of bacterial and archaeal 16S ribosomal RNA gene (*rrs*) V4 region were prepared following the “16S metagenomics sequencing library preparation” protocol (Illumina). The amplicon PCR reaction was done in a total volume of 25 µl, containing 15 ng of template DNA, 2x Kapa HiFi HotStart ReadyMix (Roche, Kapa Biosytems Ltd, South Africa) and 5 µM of each primer CapNexV4 F and CapNexV4 R (**Table S4**). Amplification was performed in a BIORAD (BIORAD, Singapore) thermal cycler with the following program: initial denaturation at 95 °C for 3 min; 25 cycles of denaturation at 95 °C for 30 s, annealing at 55 °C for 30 s and extension at 72 °C for 30 s. The final extension was performed at 72 °C for 5 min. The amplification products were purified with a QIAquick PCR purification kit (Qiagen, Germany) and the correct size of the amplicon was verified with electrophoresis in a 2 % agarose gel. The Nextera XT indexes (Illumina, San Diego, CA, USA) were added in 50 µl reaction volume containing 25 µl of 2 x KAPA HiFi HotStart ReadyMix, 5 µl of Nextera XT Index 1 and Index 2 primers, 10 µl of PCR grade water and 5 µl of the purified amplicon. The thermal cycler conditions were as follows: initial denaturation at 95 °C for 3 min followed by 8 cycles of 95 °C for 30 s, 55 °C for 30 s, 72 °C for 30 s and a final extension at 72 °C for 5 min. The library was purified with QIAquick PCR purification kit (Qiagen, Germany), size and purity were determined with Bioanalyzer DNA 1000 chip (Agilent Technologies, USA) and the concentration of the 1:10 diluted library was measured with Qubit dsDNA HS kit (Thermo Fischer Scientific, Life Technologies Corporation, Oregon, USA).

The community composition of ciliate protozoa was assessed by sequencing 18S rRNA gene. The amplicon PCR reaction was done in total volume of 25 µl, containing 15 ng of template DNA, 2x Kapa HiFi HotStart ReadyMix (Roche, Kapa Biosytems Ltd, South Africa) and 5 µM of each primer ILNA_316F and ILNA_539R (**Table S4**). Amplification was performed in a BIORAD c1000 Touch (BIORAD, Singapore) thermal cycler with a following program: initial denaturation at 95 °C for 5 min; 35 cycles of denaturation at 95 °C for 30 s, annealing at 60 °C for 30 s and extension at 72 °C for 30 s. Final extension was done at 72 °C for 5 min. The amplicon was purified with a mix of 1U of Exonuclease I (Thermo Scientific, Lithuania) and 10 U of FastAP (Thermo Scientific, Lithuania) per 5 µl of PCR product by incubating in 37 °C for 15 min and deactivating the enzymes in 85 °C for 15 min. The correct size of the amplicon was verified with electrophoresis in a 1 % agarose gel. The Nextera XT indexes (Illumina, San Diego, CA, USA) were added in 25 µl reaction volume containing 12.5 µl of 2 x KAPA HiFi HotStart ReadyMix (Roche, Kapa Biosytems Ltd, South Africa) 2.5 µl of Nextera XT Index 1 and Index 2 primer each, 5 µl of PCR grade water and 2.5 µl of purified amplicon. The thermal cycler conditions were as follows: initial denaturation at 95 °C for 3 min followed by 8 cycles of 95 °C for 30 s, 55 °C for 30 s, 72 °C for 30 s and final extension at 72 °C for 5 min. The library was then purified with QIAquick PCR purification kit (Qiagen, Germany). The size and purity were determined with Bioanalyzer DNA 1000 chip (Agilent Technologies, USA) and the concentration of the library was measured with Qubit dsDNA HS kit (Thermo Fischer Scientific, Life Technologies Corporation, Oregon, USA).

Fungal community composition was assessed by sequencing the Internal transcribed spacer 1 (ITS1) region. The amplification of ITS1 region was performed in 25 µl volume, containing 20 ng of DNA, 1 x Dream Taq buffer (Thermo Fischer Scientific, Lithuania) 0.2 mM of dNTP mix (Thermo Fischer Scientific, Lithuania), 1mM of MgCl_2,_ 0.5 U of DreamTaq DNA polymerase (Thermo Fischer Scientific, Lithuania), 0.5 µM of each primer ILNA Neo 18S For and ILNA Neo 5.8S Rev (**Table S4**). The thermal cycler conditions were as follows: initial denaturation at 95 °C for 3 min; 35 cycles of denaturation at 95 °C for 30 s, annealing at 59 °C for 30 s and extension at 68 °C for 30 s. Final extension was done at 68 °C for 5 min. The amplification products were purified with a QIAquick PCR purification kit (Qiagen, Germany) and the quality and size of the product was checked with electrophoresis on a 1 % agarose gel. The Nextera XT indexes were attached as described above for the ciliate library. Concentration of the library was measured with Qubit dsDNA HS kit (Thermo Fischer Scientific, Life Technologies Corporation, Oregon, USA), and the size and purity was determined with Bioanalyzer DNA 1000 chip (Agilent Technologies, USA). Samples were diluted to 4nM concentration and pooled. Libraries were sequenced with Illumina MiSeq (Finnish Functional Genomics Centre, Turku) using PE approach with 2 x 250 bp chemistry for 16S rRNA gene library, and 2 x 300 bp for ciliate protozoa 18S rRNA gene library and fungal ITS1 library.

## Quantification of bacterial communities

### Preparation of standard curves

DNA extracted from *Prevotella ruminicola* (DSM 11371) was used as a template for standard curve preparation. The 16S ribosomal RNA gene (*rrs*) was amplified using 8-27F and 1512-1492R primers (**Table S4**). The concentration of the amplicon was measured with a spectrophotometer and the correct size of the amplicon (1492 bp) was verified with electrophoresis in a 2 % agarose gel.

The number of copies was calculated with a formula,

$$\frac{6.02 \times{10}^{23}\times DNA quantity}{660 \times amplicon size bp}$$

Where 6.02 × 10^23^ is the Avogadro constant, DNA quantity is the measured Nanodrop concentration in g/µl, 660 is the molecular mass of the nucleotide, and the amplicon size is the estimated number of nucleotides in the amplicon (1492 bp). The amplicon was then diluted to a concentration of 5×10^9^ and further diluted in a 10 base logarithmic series until 5×10^2^ copies/µl. From each dilution 2 µl of DNA was used in creating standard curve, giving a logarithmic scale of number of copies from 10^3^ to 10^9^. Threshold cycle of each dilution was measured by amplifying the V4 area (279 bp) with a second set of primers 520F and 799r2cor (**Table S4**) in a StepOne thermocycler (Applied Biosystems, Villebon sur Yvette, France). The results were regressed against the logarithmic scale concentration to achieve the standard curve. Amplification reaction (20 µl) contained 0.75 x TB Green Premix Ex Taq II (TaKaRa Bio inc., China), 0.5 µM primers each and 2µl of each standard DNA. The thermocycler conditions were as follows: initial denaturation in 95°C for 30 s, 40 cycles of denaturing at 95°C for 15 s and annealing at 60°C for 30 s; melt curve with denaturation at 95°C for 15 s, annealing at 65°C for 15 s and denaturation at 95°C for 15 s with a ramp incline of +0.4°C. The effectiveness and the quality of the standard curve were checked, and information of the standard curve saved. Two points from the standard curve (10^5^ and 10^7^ copies per reaction) were aliquoted to smaller fractions and frozen to -80°C to be used as an inter-plate control.

### Quantification of bacteria in rumen samples

The threshold cycle of each sample was measured by amplifying the 16S rRNA gene V4 area (279 bp) with primers 520F and 799r2cor (**Table S4**) in a StepOnePlus thermocycler (Applied Biosystems, Villebon sur Yvette, France). Amplification reaction (20 µl) contained 0.75 x TB Green Premix Ex Taq II (TaKaRa Bio inc., China), 0.5 µM primers each and 20 ng of rumen liquid DNA. The thermocycler conditions were as follows: initial denaturation at 95°C for 30 s, 40 cycles of denaturing at 95°C for 15 s and annealing at 60°C for 30 s; melt curve with denaturation at 95°C for 15 s, annealing at 65°C for 15 s and denaturation at 95°C for 15 s with a ramp incline of +0.4°C. The results were regressed against the logarithmic scale concentration to achieve the standard curve and sample quantities.

## Quantification of archaeal communities in rumen samples

A mixed community rumen liquid DNA extracted from an adult cow was used as a template for standard preparation. Preparation of standard curve was done with a nested qPCR. Archaeal 16S rRNA gene fragment 1050 bp in size was amplified with primers 344F and 1406R (**Table S4**). The correct size of the amplicon was verified with electrophoresis in a 2 % agarose gel and the concentration of DNA was measured with Qubit 4 Fluorometer (Invitrogen by Thermo Fischer Scientific, Life Technologies Holdings Ltd, Singapore) with Qubit^®^ dsDNA HS Assay (Invitrogen by Thermo Fischer Scientific, Life Technologies Corporation, Oregon, USA). The amplicon copy number was calculated as described above and diluted to 5 × 10^9^ copies / µl and a serial ten-fold dilution was made to achieve seven standard points in a range of 5 × 10^8^-10^2^ copies/ µl.

A qPCR was performed to determine the archaeal community quantity in the rumen sample. A smaller area (510 bp) of the 16S rRNA gene was amplified with 896F and 1406R primers (**Table S4**). The standard curve with seven points in a range of 10^9^-10^3^ copies per well was run on each plate, and the efficiency of the run was confirmed from the standard curve. Reaction was carried out in 10 µl volume on a 384 –well plate, with 1 × Power SYBR green PCR master mix (Applied Biosystems by Thermo Fischer Scientific, Life Technologies Ltd, UK), 0.15 µM of each primer, 10 ng of template DNA or 2 µl of each standard dilution. The conditions of the thermal cycler (Viia7, Applied Biosystems) were as follows: initial denaturation and enzyme activation at 95°C for 10 min; 40 cycles of denaturing at 95°C for 15 s, annealing at 60°C for 1 min, melt curve stage with denaturation at 95°C for 15 s, annealing at 65°C for 15 s and final denaturation at 95°C for 15 s with a ramp increment of 0.4°C. All standards and samples were analyzed with three replicates.

## Quantification of ciliate protozoa communities in rumen samples

A mixed community rumen liquid DNA extracted from an adult cow was used as a template for standard preparation. Preparation of standard curve was done with a nested qPCR. Ciliate protozoan 18S rRNA gene fragment 1694 bp in size was amplified with primers P.SSU-57F and P.SSU-1747R (**Table S4**). The correct size of the amplicon was verified with electrophoresis in a 1.5 % agarose gel, excised from the gel, purified with QIAquick gel extraction kit (Qiagen, Germany) and the concentration of DNA was measured with Qubit 4 Fluorometer (Invitrogen by Thermo Fischer Scientific, Life Technologies Holdings Ltd, Singapore) with Qubit^®^ dsDNA HS Assay (Invitrogen by Thermo Fischer Scientific, Life Technologies Corporation, Oregon, USA). The amplicon copy number was calculated as described above and diluted to 5 × 10^9^ copies / µl and a serial ten-fold dilution was made to achieve standard points in a range of 5 × 10^8^-10^2^ copies/ µl.

A qPCR was performed to determine the ciliate protozoa community quantity in the rumen sample. A smaller area (223 bp) of the 18S rRNA gene was amplified with 316F and 539R primers (**Table S4**). The standard curve with six points in a range of 10^8^-10^3^ copies per well was run on each plate, and the efficiency of the run was confirmed from the standard curve. Reaction was carried out in 10 µl volume on a 384 –well plate, with 1 × Power SYBR green PCR master mix (Applied Biosystems by Thermo Fischer Scientific, Life Technologies Ltd, UK), 0.25 µM of each primer, 10 ng of template DNA or 2 µl of each standard dilution. The conditions of the thermal cycler (Viia7, Applied Biosystems) were as follows: initial denaturation and enzyme activation at 95°C for 10 min; 40 cycles of denaturing at 95°C for 30 s, annealing at 60°C for 30 s and extension at 72°C for 30 s, melt curve stage with denaturation at 95°C for 15 s, annealing at 60°C for 1 min and final denaturation at 95°C for 15 s with a ramp increment of 0.4°C. All standards and samples were analyzed with three replicates.

## Quantification of anaerobic fungal communities of rumen samples

A mixed community rumen liquid DNA extracted from an adult cow was used as a template for standard preparation. Preparation of standard curve was done with a nested qPCR. An area (c. 350 bp) containing the ITS1 region was amplified with primers Neo 18S For and Neo 5.8S Rev (**Table S4**). The correct size of the amplicon was verified with electrophoresis in a 1.5 % agarose gel, excised from the gel, purified with QIAquick gel extraction kit (Qiagen, Germany) and the concentration of DNA was measured with Qubit 4 Fluorometer (Invitrogen by Thermo Fischer Scientific, Life Technologies Holdings Ltd, Singapore) with Qubit^®^ dsDNA HS Assay (Invitrogen by Thermo Fischer Scientific, Life Technologies Corporation, Oregon, USA). The amplicon copy number was calculated as described above and diluted to 5 × 10^9^ copies / µl and a serial ten-fold dilution was made to achieve standard points in a range of 5 × 10^8^-10^1^ copies/ µl.

A qPCR was performed to determine the anaerobic fungal community quantity in the rumen sample. A smaller area (120 bp) of the ITS1 region was amplified with FungiF and FungiR primers (**Table S4**). The standard curve with seven points in a range of 10^8^-10^2^ copies per well was run on each plate, and the efficiency of the run was confirmed from the standard curve. Reaction was carried out in 15 µl volume on a 384 –well plate, with 1 × Power SYBR green PCR master mix (Applied Biosystems by Thermo Fischer Scientific, Life Technologies Ltd, UK), 2 mM of added MgCl_2,_ 1 µM of each primer, 10 ng of template DNA or 2 µl of each standard dilution. The conditions of the thermal cycler (Viia7, Applied Biosystems) were as follows: initial denaturation and enzyme activation at 95°C for 10 min; 45 cycles of denaturing at 95°C for 15 s, annealing and extension at 60°C for 1 min, melt curve stage with denaturation at 95°C for 15 s, annealing at 60°C for 1 min and final denaturation at 95°C for 15 s with a ramp increment of 0.4°C. All standards and samples were analyzed with three replicates.

## Bioinformatics

#
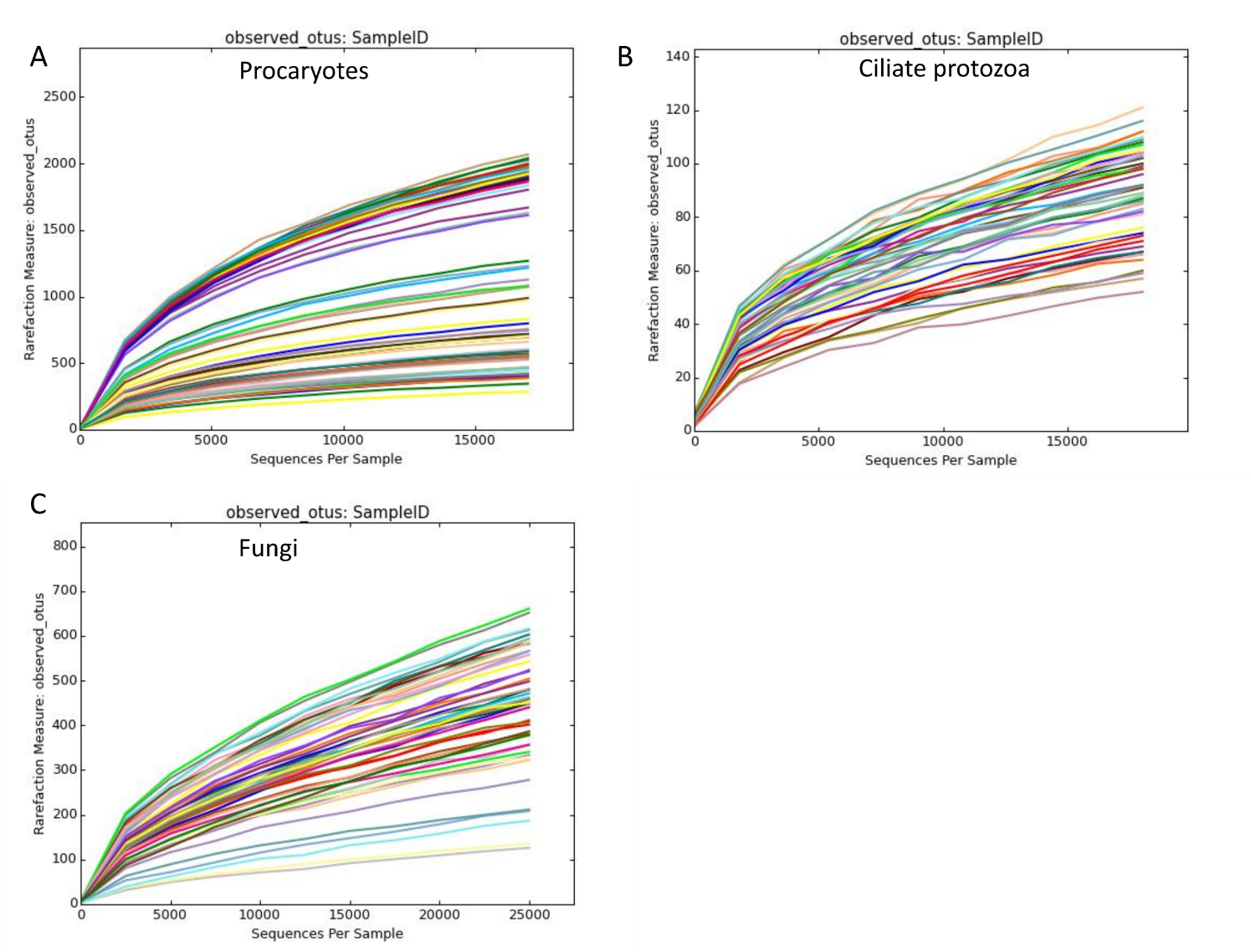


# Figure S1. Alpha rarefaction plots of observed OTUs of each sample in (A) a subsampled prokaryote sequence data (17 000 reads), (B) ciliate protozoa data (18 000 reads), and (C) anaerobic fungal data (25 000 reads).

## Statistical analyses

The rumen VFA and ammonia-N concentrations, average weekly feed consumption, growth, blood immunoglobulin G levels were analyzed using Mixed procedure in SAS version 9.4 (SAS Institute Inc., Cary, NC, USA.). Normality was confirmed with Shapiro-Wilk test. The weight, concentrate and silage consumption data were log-transformed to achieve normal distribution. The calf was treated as the experimental unit. Treatment, week and the interaction of the treatment and week were treated as fixed effects. Pair and interaction of pair and week were treated as random effects. Week was treated as a repeated effect. Based on the model fit statistics the covariance structure was set to “autoregressive” for analyzing weight, growth, daily feed consumption, bacterial and archaeal copies and to “unstructured” for analyzing blood IgG concentrations, volatile fatty acids. The effects were estimated using Residual Maximum Likelihood (REML) method and were declared significant at P ≤ 0.05. Pairwise comparisons between weeks, treatment and interaction of week and treatment were performed with TUKEY’s test. The copy numbers of ciliate protozoa 18S rRNA gene and fungal ITS1 region didn’t meet the model assumptions and the results were analyzed with Kruskal-Wallis rank sum test and pairwise comparisons with Wilcoxon rank sum test. For ciliate protozoa, only week 6 and week 8 data were used in statistical analysis.

Microbial community alpha diversity changes in T- and C-groups over 8-week period were estimated using Shannon index, Simpson index and the number of observed OTUs, as implemented in Qiime and the statistical analysis was performed with Kruskal-Wallis rank sum test and pairwise comparisons with Wilcoxon rank sum test.

To explore treatment and time effect on the changes in microbial community structure, between-sample diversity was evaluated as Bray-Curtis dissimilarities following Hellinger transformation and visualized using principal coordinate analysis (PCoA) as implemented in R packages *Microbiome* (Lahti et al., 2017) and *Phyloseq* (McMurdie and Holmes, 2013). The data was analyzed separately for comparison between calf groups and comparison between calf groups and the donor cow. The significance for the group effect of week, treatment and treatment × week was estimated using distance-based permutational multivariate analysis (Adonis) as implemented in R package *vegan* (Oksanen et al., 2019) and the pairwise comparisons between week × treatment with Permutational multivariate analysis of variance with False discovery rate adjustment with R package *RVAideMemoire* (Herve, 2020). Because C-group remained defaunated of ciliate protozoa during first four weeks, the effect of week was calculated for T-group samples separately, and the group effect of week, treatment and treatment × week was estimated for data containing only observations from weeks 6 and 8.

Treatment and time effects on individual microbial taxa in T- and C-groups were evaluated using Mixed-procedure in SAS, as described above with autoregressive covariant structure. The bacterial and fungal data was filtered to contain OTUs that were prevalent in at least 12.5 % (6 samples). To achieve normal distribution, data were (log_2_(1+x)) transformed. Treatment and time effects on bacterial genera and fungal species were evaluated using ANOVA and pairwise comparisons were done with TUKEY’s test in base R. To explore the differences in core microbial communities during different stages of development, core microbiome for T- and C-groups were calculated separately for all four sampling weeks by including only OTUs that were present in all individuals during each week. These core OTUs were compared between groups and between OTUs found in donor cow. Due to small core community size, the archaea core community results are presented with bacterial core community.

## References

Herve, M. (2020). Package “RVAideMemoire”. Version 0.9-75. Available at: https://cran.r-project.org/web/packages/RVAideMemoire/RVAideMemoire.pdf.

Lahti, L., Sudarshan, S., and Al, E. (2017). Tools for microbiome analysis in R. Version 1.9.97. Available at: https://microbiome.github.io/tutorials/.

McMurdie, P. J., and Holmes, S. (2013). phyloseq: An R Package for Reproducible Interactive Analysis and Graphics of Microbiome Census Data. *PLoS One* 8, e61217. doi:10.1371/journal.pone.0061217.

Oksanen, J., Blanchet, F. G., Friendly, M., Kindt, Roeland Legendre, P., McGlinn, D., Minchin, P. R., et al. (2019). vegan: Community Ecology Package. R package version 2.5-6. Available at: https://cran.r-project.org/package=vegan.
