# Supplementary material for: Fresh Rumen Liquid Inoculant Enhances the Rumen Microbial Community Establishment in Pre-weaned Dairy Calves
Source: Front Microbiol. 2022 Jan 12;12:758395. doi: 10.3389/fmicb.2021.758395 (PMC8790516; doi:10.3389/fmicb.2021.758395)
Supplement: Supplementary file 1 [file Data_Sheet_1.zip › Supplementary Tables S1, S2, S3.DOCX]

Table S1. The schedule and meal sizes for liquid feeding. Meal was given four times a day.

| Age  days | Colos­trum  L / dose | Milk  L / dose | Milk replacement  L / dose | Total  volume  L / day | Notes |
| --- | --- | --- | --- | --- | --- |
| 0 - 3 | 2.0 |  |  | 8.0 | The 1^st^ dose a mixture from 3 cows, frozen in n -20 ºC. The 2^nd^ dose was colostrum from the first milking of own dam. Thereafter, bulk colostrum. |
| 4 - 7 |  | 2.0 |  | 8.0 |  |
| 8 - 9 |  | 1.5 | 0.5 | 8.0 |  |
| 10 - 11 |  | 1.0 | 1.0 | 8.0 |  |
| 12 - 28 |  |  | 2.0 | 8.0 |  |
| 29 - 35 |  |  | 2.0 and 1.5 | 7.0 | Morning and evening feeding 2.0 L; day and afternoon feeding 1.5 |
| 36 - 42 |  |  | 1.5 | 6.0 |  |
| 43 - 49 |  |  | 1.5 and 1.0 | 5.0 | Morning and evening feeding 1.5 L; day and afternoon feeding 1.0 |
| 50-56 |  |  | 1.5 and 0.0 | 3.0 | Morning and evening feeding 1.5 L; day and afternoon feeding no milk replacement |

Table S2. Formulation of feeds offered to animals during the experiment.

| **Concentrate** | **Milk replacer** | **Silage** | **Hay** |
| --- | --- | --- | --- |
| Field mustard (*Brassica rapa oleifera*) | Skim milk powder | ~65% Timothy grass (*Phleum pretense*) |  |

| Wheat (*Triticum aestivum*) | Oil-Vitamin- and micronutrient premix (Palm oil, coconut oil) | ~35% Meadow fescue (*Festuca pratensis*) |  |
| --- | --- | --- | --- |
| Molasses sugar beet pulp | Whey powder |  |  |
| Barley (*Hordeum vulgare*) | Buttermilk powder |  |  |
| Fava bean (*Vicia faba*) |  |  |  |
| Beet molasses |  |  |  |
| Wheat bran |  |  |  |
| Rumen protected fat |  |  |  |
| Calcium carbonate |  |  |  |
| Ground soy |  |  |  |
| Sodium chloride |  |  |  |
| Sodium bicarbonate |  |  |  |
| Magnesium oxide |  |  |  |
| Organic micronutrient mixture |  |  |  |
| Mono calcium phosphate |  |  |  |

Table S3. Description of nutritional values of the feeds.

| **Macronutrients %** | **Concentrate** | **Milk replacer *** | **Silage** | **Hay** |
| --- | --- | --- | --- | --- |
|  |  |  |  |  |
| Crude protein | 20.1 | 20.0 | 16.2 | 13.0 |
| Crude fat | 5.3* | 17.0 |  | 3^1^ |
| Crude fiber | 7.0* | 0.02 |  | 35^1^ |
| Neutral detergent fiber NDF | 20.1 |  | 50.8 | 63.0 |
| Ash | 7.7 | 7.0 | 7.0 | 4.1 |
| Sugars g/kg DM |  |  | 10.6 | 9.1 |
| Starch g/kg DM | 25.4 |  |  |  |
| Ca % |  | 1.0 |  |  |
| P % |  | 0.8 |  |  |
| Mg % |  | 0.1 |  |  |
| Na % |  | 0.5 |  |  |
| Dry matter g/kg | 874 | 970^1^ | 258 | 860^1^ |
| Metabolizable protein g/kg DM | 120.0 |  |  |  |
| Protein balance in the rumen g/kg DM | 46.0 |  | 35^1^ | 12^1^ |
| Metabolizable Energy MJ/kg DM | 18.7 | 12.9^1^ | 11.0^1^ | 19.3 |
|  |  |  |  |  |
| Ca % |  | 1.0 |  |  |
| P % |  | 0.8 |  |  |
| Mg % |  | 0.1 |  |  |
| Na % |  | 0.5 |  |  |
| **Micronutrients (mg/kg)** |  |  |  |  |
| Vitamin C (3a300) IU |  | 100.0 |  |  |
| Vitamin E (*all-rac-alpha-tocopheryl acetate*) (3a700) | 55.0 |  |  |  |
| D-(+)-Biotin | 0.9 |  |  |  |
| Cobolt carbonate (E3) | 0.8 |  |  |  |
| Calsium iodate E2 | 4.0 | 1.3 |  |  |
| Copper | 31.0 |  |  |  |
| Cupric chelate | 9.0 |  |  |  |
| Copper (II) sulfide pentahydrate (E4) | 18.0 | 10.0 |  |  |
| Iron (II) sulfate pentahydrate (E1) |  | 125.0 |  |  |
| Selenium (premix + natural) | 0.7 |  |  |  |
| Sodium selenite (E8) | 0.3 | 0.3 |  |  |
| Selenomethionine (3b8.12) | 0.3 |  |  |  |
| Manganese | 80.0 |  |  |  |
| Manganese oxide (E5) | 26.0 |  |  |  |
| Manganese chelate (E5) | 13.0 |  |  |  |
| Mangan(II)sulfate monohydrate (E5) |  | 40.0 |  |  |
| Zinc (premix+natural) | 118.0 |  |  |  |
| Zinc chelate (E6) | 25.0 |  |  |  |
| Zinc Oxide (E6) | 50.0 |  |  |  |
| Zinc sulfate (E6) |  | 100.0 |  |  |
|  |  |  |  |  |
| Vitamin A (3a672a) IU | 9000 | 25000 |  |  |
| Vitamin D3 (E671) IU | 3000 | 4500 |  |  |
|  |  |  |  |  |
| **Other additives**: |  |  |  |  |
| *Bacillus licheniformis* (DSM 5749) + |  | 1.28*10^9^ pmy/kg |  |  |
| *Bacillus subtilis (*DSM 5750) 1/1 |  |  |  |  |
| *Enterococcus faecium* (NCIMB 11181) |  | 2.5 * 10^9^ pmy/kg |  |  |

*Nutritional composition is provided by the manufacturer.

^1^Natural Resources Institute Finland, 2020. Feed tables and feeding recommendations. Accessed Sep 15, 2020. [www.luke.fi/feedtables](http://www.luke.fi/feedtables)
