# Supplementary material for: Fresh Rumen Liquid Inoculant Enhances the Rumen Microbial Community Establishment in Pre-weaned Dairy Calves
Source: Front Microbiol. 2022 Jan 12;12:758395. doi: 10.3389/fmicb.2021.758395 (PMC8790516; doi:10.3389/fmicb.2021.758395)
Supplement: Supplementary file 1 [file Data_Sheet_1.zip › Table S4.docx]

**Table S4.** Primer pairs used for the quantification of rumen microbial communities and for the preparation of sequencing libraries.

| **Target organism** |  | **Primer** | **Sequence 5'-3'** | **Target area** | **Amplicon size bp** | **Reference** |
| --- | --- | --- | --- | --- | --- | --- |
| **qPCR standard preparation** | | | | | | |
| Bacteria | F | F8-27 | AGAGTTTGATCCTGGCTCAG | 16S | 1485 | Turnbaugh et al. (2009) |
| Bacteria | R | 1512R | GNTACCTTGTTACGACTT |  |  |  |
| Archaea | F | 344F | ACGGGGYGCAGCAGGCGCGA | 16S | 1062 | Ohene-Adjei et al. (2007) |
| Archaea | R | 1406R | ACGGGCGGTGTGTGCAAG |  |  | Loy et al. (2002) |
| Ciliate protozoa | F | P.SSU-57F | CAYGTCTAAGTATAAATAACTAC | 18S | 1694 | Sylvester et al. (2004) |
| Ciliate protozoa | R | P.SSU-1747R | CTCTAGGTGATWWGRTTTAC |  |  |  |
| Fungi | F | Neo 18S For | AAT CCT TCG GAT TGG CT | ITS1 | 350-450 | Edwards et al. (2008) |
| Fungi | R | Neo 5.8S Rev | CGA GAA CCA AGA GAT CCA |  |  |  |
| **Quantitation with qPCR** | | | | | | |
| Bacteria | F | 520F | AGCAGCCGCGGTAAT | 16S V4 | 280 | Edwards et al. (2007) |
| Bacteria | R | 799r2cor | CAGGGTATCTAATCCTGTT |  |  |  |
| Archaea | F | 896F | AGGAATTGGCGGGGGAGCAC | V6-V8 | 510 | Stahl and Amann (1991)  Ohene-Adjei et al. (2007) |
| Archaea | R | 1406R | ACGGGCGGTGTGTGCAAG |  |  | Loy et al. (2002) |
| Ciliate protozoa | F | 316F | GCTTTCGWTGGTAGTGTATT | 18S | 223 | Sylvester et al. (2004) |
| Ciliate protozoa | R | 539R | CTTGCCCTCYAATCGTWCT |  |  |  |
| Fungi | F | FungiF | GAGGAAGTAAAAGTCGTAACAAGGTTTC | 18S-ITS1 | 120 | Denman & McSweeney (2006) |
| Fungi | R | FungiR | CAAATTCACAAAGGGTAGGATGATT |  |  |  |
| **Amplicon sequencing** | | | | | | |
| Bacteria & archaea | F | CapNexF (515F) | TCGTCGGCAGCGTCAGATGTGTATAAGAGACAGGTGCCAGCMGCCGCGGTAA | 16S V4 | 290 | Caporaso et al. (2011) |
| Bacteria & archaea | R | CapNexR (806R) | GTCTCGTGGGCTCGGAGATGTGTATAAGAGACAGGGACTACHVGGGTWTCTAAT |  |  |  |
| Ciliates | F | ILNA_316F | TCGTCGGCAGCGTCAGATGTGTATAAGAGACAGGCTTTCGWTGGTAGTGTATT | 18S V3 (SR1) | 225 | Sylvester et al. (2004) |
| Ciliates | R | ILNA_539R | GTCTCGTGGGCTCGGAGATGTGTATAAGAGACAGCTTGCCCTCYAATCGTWCT |  |  |  |
| Fungi | F | ILNA_Neo18SF | TCGTCGGCAGCGTCAGATGTGTATAAGAGACAGAAT CCT TCG GAT TGG CT | ITS1 | 350-450 | Edwards et al. (2008) |
| Fungi | R | ILNA_Neo5.8SR | GTCTCGTGGGCTCGGAGATGTGTATAAGAGACAGCGA GAA CCA AGA GAT CCA |  |  |  |

**References**

Caporaso, J. G., Lauber, C. L., Walters, W. A., Berg-lyons, D., Lozupone, C. A., Turnbaugh, P. J., et al. (2011). Global patterns of 16S rRNA diversity at a depth of millions of sequences per sample. *PNAS* 108, 4516–4522. doi:10.1073/pnas.1000080107/-/DCSupplemental.www.pnas.org/cgi/doi/10.1073/pnas.1000080107.

Denman, S. and McSweeney, C. (2006). Development of a real-time PCR assay for monitoring anaerobic fungal and cellulolytic bacterial populations within the rumen. *FEMS Microbiol. Ecol.* 58, 572-582. doi: 10.1111/j.1574-6941.2006.00190.x

Edwards, J. E., Huws, S. A., Kim, E. J., and Kingston-Smith, A. H. (2007). Characterization of the dynamics of initial bacterial colonization of nonconserved forage in the bovine rumen. *FEMS Microbiol. Ecol.* 62, 323–335. doi:10.1111/j.1574-6941.2007.00392.x.

Edwards, J. E., Kingston-Smith, A. H., Jimenez, H. R., Huws, S. A., Skøt, K. P., Griffith, G. W., et al. (2008). Dynamics of initial colonization of nonconserved perennial ryegrass by anaerobic fungi in the bovine rumen. *FEMS Microbiol. Ecol.* 66, 537–545. doi:10.1111/j.1574-6941.2008.00563.x.

Loy, A., Lehner, A., Lee, N., Adamczyk, J., Meier, H., Ernst, J., et al. (2002). Oligonucleotide Microarray for 16S rRNA Gene-Based Detection of All Recognized Lineages of Sulfate-Reducing Prokaryotes in the Environment. *Appl. Environ. Microbiol.* 68, 5064–5081. doi:10.1128/AEM.68.10.5064.

Ohene-Adjei, S., Teather, R. M., Ivan, M., and Forster, R. J. (2007). Postinoculation protozoan establishment and association patterns of methanogenic archaea in the ovine rumen. *Appl. Environ. Microbiol.* 73, 4609–4618. doi:10.1128/AEM.02687-06.

Stahl, D., and Amann, R. (1991). “Development and application of nucleic acid probes.,” in *Nucleic Acids Techniques in Bacterial Systematics*, eds. E. Stackebrandt and M. Goodfellow (Chichester: John Wiley & Sons,), 205–248.

Sylvester, J. T., Karnati, S. K. R., Yu, Z., Morrison, M., and Firkins, J. L. (2004). Development of an Assay to Quantify Rumen Ciliate Protozoal Biomass in Cows Using Real-Time PCR. *Nutr. Methodol.* 134, 3378–3384. doi:doi: 10.1093/jn/134.12.3378.

Turnbaugh, P. J., Hamady, M., Yatsunenko, T., Cantarel, B. L., Duncan, A., Ley, R. E., et al. (2009). A core gut microbiome in obese and lean twins. *Nature* 457, 480–484. doi:10.1038/nature07540.
