# Supplementary material for: Fresh Rumen Liquid Inoculant Enhances the Rumen Microbial Community Establishment in Pre-weaned Dairy Calves
Source: Front Microbiol. 2022 Jan 12;12:758395. doi: 10.3389/fmicb.2021.758395 (PMC8790516; doi:10.3389/fmicb.2021.758395)
Supplement: Supplementary file 1 [file Data_Sheet_1.zip › Table S5.DOCX]

**Table S5**. The feed intake of calves receiving rumen liquid inoculum (T-group) and control calves (C-group) during the eight-week treatment period. The data are presented as least squares means (LS mean) ± standard error (SE), and statistical significance of Treatment (Trt), Week (Wk) and interaction (Trt x Wk). Different letters indicate statistically significant differences in pairwise comparison between groups by week (Tukey-Kramer adjusted).

|  |  |  | **Feed intake** |  |  |  |
| --- | --- | --- | --- | --- | --- | --- |
| **Study week** |  |  | Milk/milk replacer  Kg/day | Concentrate  g DM d | Silage  g DM d |  |
| Birth | T-group |  |  |  |  |  |
|  | C-group |  |  |  |  |  |
|  |  |  |  |  |  |  |
| Week 1 | T-group |  | 6.7^c^ | 0.7^h^ |  |  |
|  | C-group |  | 6.5^cd^ | 1.3^h^ |  |  |
|  |  |  |  |  |  |  |
| Week 2 | T-group |  | 7.0^c^ | 9.3^gh^ |  |  |
|  | C-group |  | 7.0^c^ | 4.8^g^ |  |  |
|  |  |  |  |  |  |  |
| Week 3 | T-group |  | 7.6^ab^ | 26.8^def^ |  |  |
|  | C-group |  | 7.6^ab^ | 5.8^g^ |  |  |
|  |  |  |  |  |  |  |
| Week 4 | T-group |  | 7.7^a^ | 29.9^def^ |  |  |
|  | C-group |  | 7.7^a^ | 8.0^fg^ |  |  |
|  |  |  |  |  |  |  |
| Week 5 | T-group |  | 7.0^bc^ | 46.1^bcde^ |  |  |
|  | C-group |  | 7.0^bc^ | 20.2^ef^ |  |  |
|  |  |  |  |  |  |  |
| Week 6 | T-group |  | 6.0^d^ | 104.5^bc^ |  |  |
|  | C-group |  | 6.0^d^ | 35.6 ^cde^ |  |  |
|  |  |  |  |  |  |  |
| Week 7 | T-group |  | 5.0^e^ | 78.7^bcd^ | 132.4 |  |
|  | C-group |  | 5.0^e^ | 115.4^b^ | 79.3 |  |
|  |  |  |  |  |  |  |
| Week 8 | T-group |  | 3.0^f^ | 606.3^a^ | 164.6 |  |
|  | C-group |  | 3.1^f^ | 392.8^a^ | 86.6 |  |
| **Standard error** |  |  | 2.2 | 1.9 | 1.5 |  |
| **Statistics** |  |  |  |  |  |  |
| Wk | P-value |  | 0.0001 | 0.0001 | 0.421 |  |
| Trt | P-value |  | 0.730 | 0.076 | 0.324 |  |
| Trt × Wk | P-value |  | 0.901 | 0.176 | 0.689 |  |
