# Supplementary material for: Fresh Rumen Liquid Inoculant Enhances the Rumen Microbial Community Establishment in Pre-weaned Dairy Calves
Source: Front Microbiol. 2022 Jan 12;12:758395. doi: 10.3389/fmicb.2021.758395 (PMC8790516; doi:10.3389/fmicb.2021.758395)
Supplement: Supplementary file 1 [file Data_Sheet_1.zip › Table S7.DOCX]

**Table S7.** The relative abundance of rumen bacterial phyla and archaea in calves receiving rumen liquid inoculum (T-group) and control calves (C-group) during eight-week experimental period. The taxa that occur in less than six samples are grouped into “Other rare taxa”. Results are presented as averages ± standard deviation, and statistical significances of Treatment (Trt), Week (Wk) and interaction (Trt x Wk).

|  |  |  | **Week 2** | | **Week 4** | | **Week 6** | | **Week 8** | |  | **Statistics** | | | |
| --- | --- | --- | --- | --- | --- | --- | --- | --- | --- | --- | --- | --- | --- | --- | --- |
|  | **Phylum** |  | **C-group** | **T-group** | **C-group** | **T-group** | **C-group** | **T-group** | **C-group** | **T-group** | **Donor** | **Trt** | **Wk** | **Trt**  **× Wk** |  |
|  | Bacteroidetes |  | 42.71 ± 8.25 | 37.83 ± 15.36 | 44.43 ± 4.27 | 45.21 ± 4.5 | 40.41 ± 9.50 | 40.3 ± 12.23 | 36.8 ± 7.87 | 41.81 ± 8.72 | 32.92 ± 3.05 | 0.769 | 0.089 | 0.763 |  |
|  | Firmicutes |  | 39.97 ± 2.68 | 36.39 ± 11.71 | 42.09 ± 7.62 | 31.5 ± 7.28 | 30.76 ± 7.46 | 29.2 ± 4.37 | 35.42 ± 4.44 | 34.79 ± 4.43 | 37.93 ± 3.24 | **0.002** | 0.114 | 0.361 |  |
|  | Verrucomicrobia |  | 4.24 ± 4.48 | 3.66 ± 7.83 | 0.41 ± 0.68 | 0.59 ± 0.69 | 0.40 ± 0.38 | 3.82 ± 4.34 | 1.26 ± 2.18 | 0.48 ± 0.78 | 0.94 ± 0.37 | 0.661 | 0.422 | 0.081 |  |
|  | Proteobacteria |  | 3.84 ± 4.25 | 3.27 ± 2.30 | 2.89 ± 3.01 | 6.42 ± 4.7 | 9.63 ± 3.87 | 2.32 ± 1.24 | 9.94 ± 6.08 | 5.45 ± 3.19 | 0.60 ± 0.31 | 0.184 | 0.307 | 0.189 |  |
|  | Actinobacteria |  | 2.04 ± 2.52 | 8.19 ± 12.88 | 1.80 ± 1.26 | 2.78 ± 2.61 | 3.13 ± 2.28 | 5.28 ± 5.36 | 1.68 ± 2.13 | 4.36 ± 4.77 | 2.80 ± 0.80 | 0.115 | 0.593 | 0.855 |  |
|  | Tenericutes |  | 0.23 ± 0.13 | 0.64 ± 0.84 | 0.18 ± 0.26 | 0.43 ± 0.26 | 0.57 ± 0.38 | 0.44 ± 0.38 | 0.77 ± 0.45 | 0.42 ± 0.43 | 0.79 ± 0.21 | 0.997 | 0.568 | 0.163 |  |
|  | Spirochaetes |  | 0.03 ± 0.04 | 1.63 ± 1.75 | 0.37 ± 0.70 | 1.00 ± 0.24 | 2.21 ± 1.53 | 1.11 ± 1.05 | 2.22 ± 0.82 | 0.89 ± 0.71 | 0.63 ± 0.15 | **0.007** | **< 0.001** | **< 0.001** |  |
|  | Fusobacteria |  | 0.01 ± 0.01 | 0.03 ± 0.04 | 0.02 ± 0.06 | 0.04 ± 0.06 | 0.04 ± 0.09 | 0.05 ± 0.09 | 0 ± 0.00 | 0.02 ± 0.02 | 0 ± 0.00 | 0.053 | 0.721 | 0.989 |  |
|  | Synergistetes |  | 0 ± 0.01 | 0.02 ± 0.03 | 0.20 ± 0.26 | 0.08 ± 0.08 | 0.08 ± 0.03 | 0.06 ± 0.07 | 0.03 ± 0.02 | 0.03 ± 0.03 | 0.02 ± 0.01 | 0.622 | **0.015** | 0.510 |  |
|  | TM7 |  | 0 ± 0.00 | 0 ± 0.01 | 0.04 ± 0.06 | 0.49 ± 0.55 | 0.15 ± 0.16 | 0.48 ± 0.56 | 0.26 ± 0.18 | 0.21 ± 0.27 | 1.10 ± 0.18 | 0.532 | **0.004** | 0.156 |  |
|  | Cyanobacteria |  | 0 ± 0.00 | 0 ± 0.00 | 0.06 ± 0.09 | 0.18 ± 0.20 | 0.31 ± 0.49 | 0.7 ± 0.81 | 0.67 ± 0.51 | 0.51 ± 0.50 | 0.50 ± 0.27 | 0.865 | **< 0.001** | 0.690 |  |
|  | SR1 |  | 0 ± 0.00 | 0 ± 0.00 | 0 ± 0.00 | 0.16 ± 0.19 | 0.27 ± 0.66 | 0.65 ± 0.73 | 0.14 ± 0.29 | 0.15 ± 0.19 | 0.61 ± 0.31 | 0.142 | 0.109 | 0.158 |  |
|  | Chloroflexi |  | 0 ± 0.00 | 0.01 ± 0.01 | 0.11 ± 0.2 | 0.15 ± 0.19 | 0.49 ± 0.41 | 0.23 ± 0.33 | 0.06 ± 0.04 | 0.05 ± 0.07 | 0.25 ± 0.12 | 0.703 | 0.055 | 0.315 |  |
|  | Elusimicrobia |  | 0 ± 0.00 | 0 ± 0.00 | 0 ± 0.00 | 0.23 ± 0.23 | 0.13 ± 0.16 | 0.28 ± 0.37 | 0.04 ± 0.06 | 0.08 ± 0.15 | 0.02 ± 0.02 |  |  |  |  |
|  | Fibrobacteres |  | 0 ± 0.00 | 1.73 ± 2.66 | 0 ± 0.00 | 1.29 ± 0.92 | 2.32 ± 1.76 | 3.48 ± 3.19 | 1.06 ± 0.97 | 2.09 ± 1.40 | 0.63 ± 0.19 | **< 0.001** | **< 0.001** | **< 0.001** |  |
|  | Lentisphaerae |  | 0 ± 0.00 | 0 ± 0.00 | 0 ± 0.01 | 0.05 ± 0.08 | 0.14 ± 0.24 | 0.12 ± 0.13 | 0.01 ± 0.02 | 0.02 ± 0.03 | 0.05 ± 0.03 |  |  |  |  |
|  | Planctomycetes |  | 0 ± 0.00 | 0 ± 0.00 | 0.1 ± 0.12 | 0.06 ± 0.03 | 0.06 ± 0.06 | 0.05 ± 0.03 | 0.06 ± 0.05 | 0.04 ± 0.04 | 0.17 ± 0.06 | 0.866 | **< 0.001** | 0.863 |  |
|  | Archaea |  | 0.52 ± 0.33 | 0.9 ± 0.74 | 1.51 ± 0.67 | 1.65 ± 0.32 | 1.33 ± 0.34 | 1.5 ± 0.51 | 1.67 ± 0.96 | 1.43 ± 0.93 | 4.84 ± 0.91 | 0.583 | 0.227 | 0.691 |  |
|  | Unassigned Other |  | 0.82 ± 1.33 | 2.88 ± 2.86 | 1.28 ± 1.3 | 3.13 ± 5.47 | 1.32 ± 1.36 | 1.8 ± 1.6 | 1.25 ± 1.49 | 1.56 ± 1.92 | 0.47 ± 0.23 | **0.050** | 0.383 | 0.583 |  |
|  | Other rare taxa |  | 5.58 ± 2.15 | 2.81 ± 0.94 | 4.51 ± 1.97 | 4.55 ± 2.73 | 6.23 ± 1.88 | 8.12 ± 6.18 | 6.67 ± 3.17 | 5.63 ± 3.77 | 14.76 ± 0.65 |  |  |  |  |
